# Characterization of cancer-associated fibroblast populations that promote tertiary lymphoid structure formation in murine melanoma tumors

**DOI:** 10.3389/fimmu.2026.1857037

**Published:** 2026-07-17

**Authors:** Robert Barnes, Kara Cummings, Mirna Perusina Lanfranca, Anthony B. Rodriguez, Katarzyna Stasiak, Burkhard Ludewig, Sepideh Dolatshahi, Victor H. Engelhard

**Affiliations:** 1 Departments of Biomedical Engineering, University of Virginia, Charlottesville, VA, United States; 2 Microbiology, Immunology and Cancer Biology, University of Virginia, Charlottesville, VA, United States; 3 Carter Immunology Center, University of Virginia, Charlottesville, VA, United States; 4 Medical Research Center, Cantonal Hospital St. Gallen, St. Gallen, Switzerland

**Keywords:** cell adhesion, chemokines, CXCL13, cytokines, MHC, ScRNA-seq

## Abstract

**Introduction:**

In recent years, the clinical relevance of tertiary lymphoid structures (TLS) in cancer has become increasingly clear. However, the mechanisms that promote TLS development have remained obscure, largely because of a lack of animal models in which cause and effect studies can be performed. In this study, we used a mouse model in which TLS develop spontaneously in intraperitoneal tumors to address this issue.

**Methods:**

Cxcl13-cre-tdTomato mice were implanted with B16-OVA melanoma tumors in either intraperitoneal or subcutaneous locations to establish which cells expressed CXCL13. Single cell suspensions from tumors were analyzed by flow cytometry. To address the impact of CXCL13^+^ CAF on TLS formation, we bred Cxcl13-cre tdTomato mice to R26R-iDTR^flox^ mice, which express the diphtheria toxin receptor under the control of a floxed stop codon. The resulting Cxcl13-cre/iDTR mice were treated with diphtheria toxin beginning on day 7 after IP tumor implantation. Single cell suspensions from tumors were analyzed by flow cytometry. To characterize their functional properties, CAF in IP melanoma tumors from Cxcl13-cre/eGFP mice were collected by cell sorting, analyzed for transcript expression using scRNA-seq, clustered into groups using the Seurat package, and profiled using pathway analysis and differential gene analysis.

**Results:**

Extending earlier studies in which we showed that CXCR5-expressing B cells are essential for TLS development, we found that CXCL13, the ligand for CXCR5, were expressed exclusively by a population of cancer-associated fibroblasts (CAF), and that these CAF were essential to promote B cell accumulation in these tumors. In keeping with earlier work showing that the presence of TLS was associated with smaller tumor size, we also found that the lack of B cell accumulation was associated with larger tumor size. Using scRNA-seq, we identified 7 groups of CAF in TLS containing tumors. One of these CAF groups was substantially enriched for CXCL13-expressing cells. This population was also enriched for additional genes that could promote B cell recruitment and enable regulation by a variety of cytokines. However, other genes that we have shown or hypothesize to be important in TLS development were enriched for expression in other CAF groups.

**Conclusions:**

This work indicates that TLS development in this model depends on multiple CAF populations operating at distinct points in the developmental process. Some of these populations appear similar to, but distinct from, those defined in other human and murine tumor models. However, we also discriminated new immunologically relevant populations of CAF, particularly one expressing CXCL13, which we propose acts as a central organizer of tumor-associated TLS.

## 1 Introduction

Tertiary lymphoid structures (TLS) are ectopic aggregates of T- and B-cells and dendritic cells, juxtaposed to blood vasculature expressing peripheral node addressin (PNAd) and CCL21, molecules that are usually confined to the high endothelial venules of lymph nodes (reviewed in (1, 2). Although initially described in association with chronic infection, autoimmunity, and organ transplantation, TLS are also found in tumors. Tumor-associated TLS vary considerably in cellular composition and organization (3–6). Mature dendritic cells, T_FH_, T_reg_, and B cells with immature, naïve, activated, memory, and plasma cell phenotypes are evident to varying extents. They also contain one or more of the homeostatic chemokines CCL19, CCL21, CXCL12, and CXCL13, which organize secondary lymphoid organ microarchitecture, along with cells resembling fibroblastic reticular cells and/or follicular dendritic cells (5, 7). The presence of TLS in human cancers has commonly been associated with enhanced patient survival and clinical responses to chemo- and immunotherapies, although there are exceptions (3–6). Correlations among features of TLS and effector function in tumor infiltrating lymphocytes have suggested a model in which TLS continuously recruit naïve and central memory T- and B-cells and sustain the output of new effectors, in an environment biased for presentation of tumor-derived antigen. While it has been suggested that augmentation of tumor associated TLS could be a new strategy for cancer immunotherapy, this depends on understanding the mechanisms driving their development.

To address this issue, we and others have characterized TLS that spontaneously develop in murine tumors growing in multiple anatomic locations (8–13). We initially discovered that naïve CD8 T-cells infiltrate murine tumors and become activated effectors that can enhance tumor control (9, 14). Infiltration is mediated by PNAd and CCL21, which engage L-selectin and CCR7, respectively, and are expressed on ~5% of tumor blood vessels (9). We subsequently found that tumors in lung, liver, and peritoneal cavity develop PNAd^+^ vessel-associated intratumoral TLS, but subcutaneous (SC) tumors do not (9–11). These TLS were shown to be necessary for the action of checkpoint blockade immunotherapy in controlling tumor outgrowth (11).

We unexpectedly identified a population of Cancer Associated Fibroblasts (CAF) as key players in these TLS. Compared to SC tumors, intraperitoneal (IP) tumors had ~20X as many B-cells and ~5X as many podoplanin (PDPN)^+^ CAF relative to CD31^+^ endothelial cells, while numbers of T-cells and dendritic cells were similar (11). In IP but not SC tumors, these CAF formed a reticular network that is co-extensive with the TLS (9, 11). CAF in IP tumor TLS also expressed higher levels of VCAM-1 and ICAM-1, and lower levels of Fibroblast Activating Protein (FAP), a marker associated with pro-tumor activity (15) and poor survival in cancer patients (16), than intratumoral CAF that were outside TLS. CAF in IP tumors also expressed substantially higher levels of CCL21, CXCL13, and the B-cell survival factors BAFF and APRIL than their SC counterparts, and TLS formation depended on cells expressing CXCR5, the receptor for CXCL13 (11). Finally, we showed that TLS formation could be induced by injection of a mixture of tumor cells with FAP^neg^ CAF isolated from IP tumors into a SC site, which does not otherwise support intratumoral TLS development. Collectively, this work suggested a model in which murine tumor associated TLS development is driven by a complex interplay of T-cell, B-cell, and non-lymphoid cells that deliver signals via different TNF and Lymphotoxin receptors to CAF. These CAF, in turn, secrete chemokines that attract immune cells of the TLS to a centralized location and provide the scaffolding on which they become organized.

While this is an attractive model, voluminous work from many laboratories has established that CAF perform a wide range of functions within the tumor and are crucial executors of many pro-tumor functions associated with tissue remodeling, vascularization, and extracellular matrix (ECM) deposition (17–22). It has also become clear that many tumors contain multiple populations of CAF that carry out these functions, and additional populations that appear to promote anti-tumor immunity (23–27). Studies in humans have pointed to both T cells and CAF as sources of CXCL13 that correlate with the presence of TLS (28–37), while others have associated the presence of TLS in human tumors with subsets of CAF that differentially express CCL19 (38–41). However, no in-depth analysis of CAF that have been directly demonstrated to promote TLS development has been presented. In the current work, we have used a reporter model to identify and provide greater insight into the CAF populations present in TLS-containing B16 melanoma tumors, with a particular focus on the functions that are necessary for TLS development.

## 2 Results

### 2.1 CXCL13 expression is confined to CAF in IP B16 melanoma tumors containing TLS

**Figure 1:**
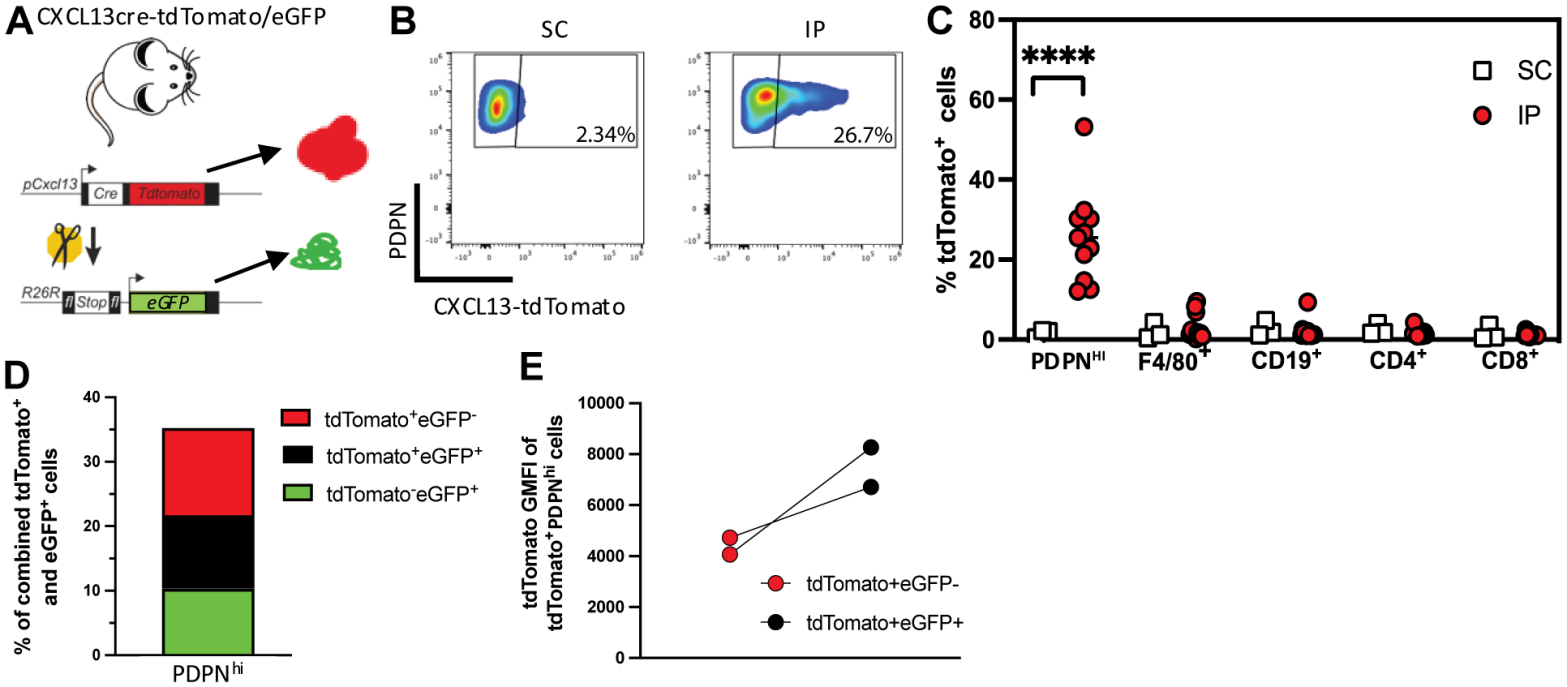
CXCL13 is selectively expressed in fibroblasts in IP tumors. Day 14 S.C. or I.P. B16-OVA tumors from Cxcl13-cre-tdTomato/R26R-eGFP^flox/flox^ mice (all homozygous for the R26R-eGFP^flox^ gene) **(A)** were prepared for flow cytometry as described in Methods. **(B)** Representative histograms of tdTomato expression in PDPN^hi^ CD31^neg^ CD45^neg^ Ter119^neg^ CAF from SC and IP tumors. **(C)** Percentage of tdTomato^+^ cells in the indicated cell populations was established using the markers described in Methods. Each data point represents an independent tumor. Results are mean ± SD analyzed by unpaired Welch’s t-test. ****, p< 0.0001. **(D)** Composite percentages of tdTomato and eGFP expression in PDPN^hi^ CD31^neg^ CD45^neg^ Ter119^neg^ CAF. **(E)** Geometric mean fluorescence (gMFI) intensities of tdTomato expression in PDPN^hi^ CD31^neg^ CD45^neg^ Ter119^neg^ CAF after gating for expression of eGFP. gMFIs were calculated on cells gated above the fluorescence minus one (FMO) control. Each line represents a single tumor.

CAF isolated from IP localized B16-OVA melanoma tumors expressed substantially elevated levels of *Cxcl13, Baff*, and *April* mRNA compared to CAF isolated from SC tumors (11) (**Supplementary Figure 1A**), and CXCL13 protein was evident in a subset of TLS-associated CAF by immunohistochemistry (**Supplementary Figure 1B**). We hypothesized that intratumoral TLS formation, and processes key to promoting intratumoral anti-tumor immunity, depended on CAF expressing these proteins, which collectively are known to promote B cell organization and survival (42, 43). To begin to test this, we bred Cxcl13-cre-tdTomato mice (44) to R26R-eGFP^flox^ mice to establish which cells in B16-OVA melanoma tumors expressed CXCL13. In the resulting F1 mice (Cxcl13-cre/eGFP), the Cxcl13 promoter drives expression of tdTomato and cre, leading to excision of a floxed stop codon and expression of eGFP (**Figure 1A**). These mice were implanted with tumors in either IP or SC locations. Tumors were isolated on day 14 and digested to create single cell suspensions. PDPN^hi^ CAF and PDPN^lo^ tumor cells were separated from CD45^+^ hematopoietic/immune cells using magnetic beads, and the resulting cell populations were stained for cell lineage markers and analyzed by flow cytometry.

Consistent with our earlier work (11), about 25% of CAF in IP tumors were tdTomato^+^, while only ~1-2% of CAF in SC tumors had upregulated this reporter, and the MFI of tdTomato was substantially lower (**Figures 1B, C**). Only very small numbers of other cell populations in these tumors expressed tdTomato, and thus, CXCL13, and these numbers were not elevated in IP vs SC tumors (**Figure 1C**). Therefore, it is extremely unlikely that these other populations contribute to the selective development of TLS in IP tumors. In addition, the mean level of tdTomato expression in the tdTomato^+^ CAF subpopulation from IP tumors was substantially higher than in the corresponding subpopulation from SC tumors (**Figure 1B**). Of the 25% tdTomato^+^ cells in IP tumors, only about half were also eGFP^+^ (**Figure 1D**). However, the level of tdTomato expression in these tdTomato^+^ eGFP^+^ CAF was over twice as high as that in tdTomato^+^ eGFP^neg^ CAF (**Figure 1E**). This suggests that a lower level of Cxcl13-cre expression is less efficient in driving recombination of genes containing floxed stop codons in cells that nonetheless express CXCL13. Intriguingly, we found that a significant fraction of PDPN^hi^ CAF from IP tumors were eGFP^+^ but tdTomato^neg^ (**Figure 1D**). This indicates that these cells expressed CXCL13 at an earlier point, leading to eGFP expression, but no longer did so at the time of tumor harvest. Collectively, these results establish that CXCL13 expression is confined to CAF in IP B16-OVA melanoma tumors at this time point, and that the Cxcl13 promoter-driven expression of cre is reasonably efficient in enabling expression of floxed molecules.

### 2.2 Cxcl13-expressing CAF drive B cell accumulation in IP B16 melanoma tumors

**Figure 2:**
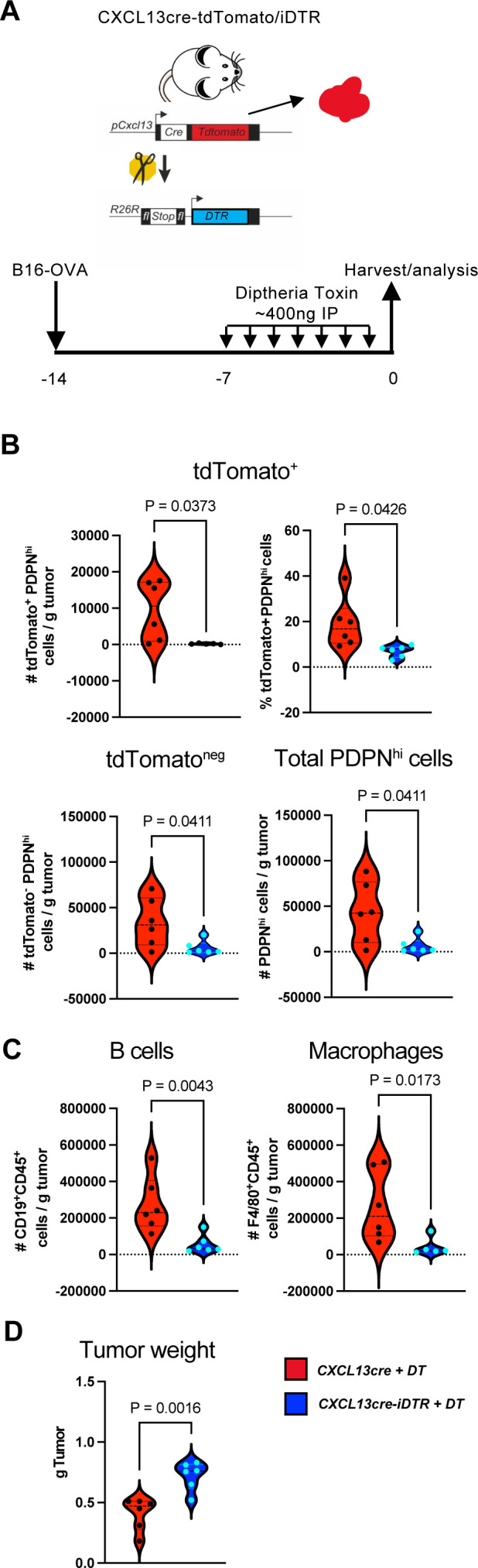
Diphtheria toxin-induced ablation of CXCL13^+^ fibroblasts in IP tumors decreases B cells and macrophages and increases tumor weight. **(A)** Cxcl13-cre-tdTomato and Cxcl13-cre-tdTomato X R26R-iDTR^flox^ mice were injected IP with B16-OVA melanoma cells. Seven days later, diphtheria toxin was administered daily for an additional 7 days, followed by tumor harvest, weighing, and preparation of cells for flow cytometry as described in Methods. **(B)** Numbers of the indicated CAF populations and their percent representation among all CAF were determined. Each data point represents an independent tumor. **(C)** Numbers of the indicated immune populations were determined. Each data point represents an independent tumor. Results are mean ± SD analyzed by unpaired Welch’s t-test. **(D)** Tumor weights were determined at the time of harvest. Each data point represents an independent tumor.

To directly address the impact of CXCL13^+^ CAF on TLS formation, we bred Cxcl13-cre tdTomato mice to R26R-iDTR^flox^ mice, which express the diphtheria toxin receptor under the control of a floxed stop codon (**Figure 2A**). Treatment of the resulting Cxcl13-cre/iDTR mice with diphtheria toxin beginning on day 7 after IP tumor implantation led to a 98% reduction in the number of tdTomato^+^ CAF per tumor gram (**Figure 2B**). This also led to an 85% reduction in the number of tdTomato^neg^ CAF, and of CAF overall. While this is in part a likely consequence of ablating the precursors of the tdTomato^neg^ eGFP^+^ subpopulation described above, the overall magnitude of reduction was substantially higher than would have been expected and therefore must also involve a reduction in the number of CAF that would be tdTomato^neg^ eGFP^neg^. Importantly, tumors from diphtheria toxin-treated Cxcl13-cre/iDTR mice also contained substantially fewer B cells and macrophages per tumor gram, and tumor size was significantly larger (**Figure 2C**). While technical issues precluded a direct demonstration that this inhibited TLS development, our earlier work (11) demonstrated that B cells are essential for TLS to form. These data demonstrate that CXCL13^+^ CAF recruit B cells into tumors, and play an important role in controlling tumor outgrowth, presumably through their impact on B cell recruitment with consequent effects on TLS formation. However, the data also suggest that B cells promote the expansion of multiple populations of CAF, not only those that directly express CXCL13, which in turn may promote the infiltration of monocytes/macrophages, pointing to a potential evolution of the tumor microenvironment that might ultimately promote, rather than inhibit, tumor outgrowth.

### 2.3 ScRNA-seq analysis reveals multiple CAF groups with distinct immunological and non-immunological profiles

To further characterize the functional properties of CAF expressing CXCL13, IP melanoma tumors were collected from Cxcl13-cre/eGFP mice, and the 4 groups of CAF identified above were collected separately by cell sorting on PDPN, tdTomato, and eGFP. Collected cells were analyzed for transcript expression using scRNA-seq. We identified 3 clusters of fibroblasts, characterized by expression of one or more collagens, and residual populations of immune and melanoma cells, characterized by expression of CD45 (*Ptprc*) and *Pax3*, respectively (**Supplementary Figure 2**). These latter clusters were removed from further analysis.

**Figure 3:**
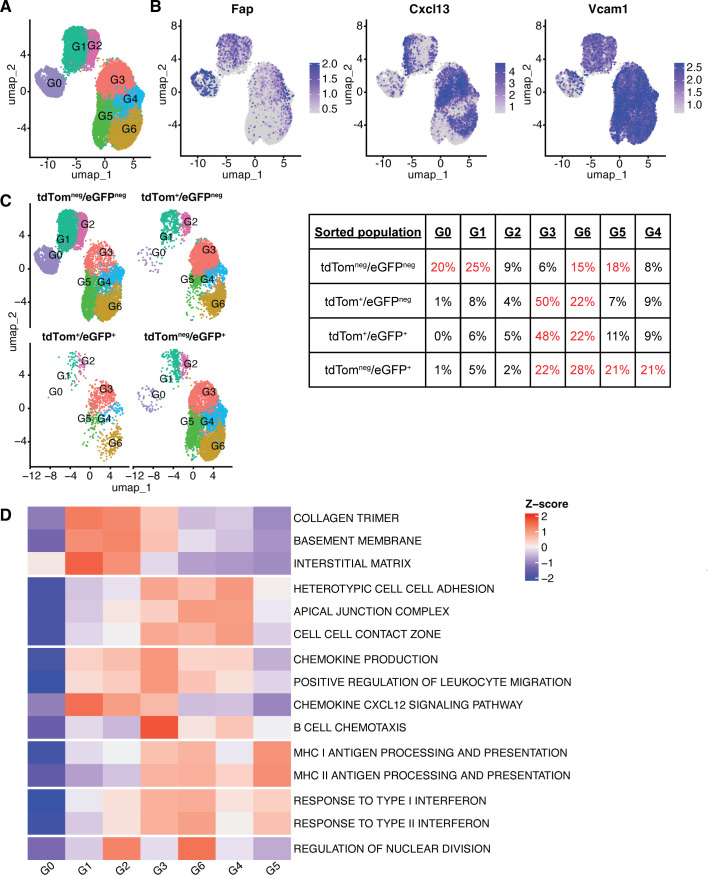
Unsupervised cluster analysis of CAF populations from IP B16 melanomas. **(A–C)** Single cell transcriptomic data of all fibroblasts was clustered by similarity in gene expression as described in Methods and plotted in a UMAP. **(A)** Distinct fibroblast groups are identified by color and group number. **(B)** Distribution of TLS-relevant CAF genes. **(C)** Distribution of individual fibroblast populations sorted for tdTomato and eGFP expression among distinct fibroblast groups. **(D)** A subset of differentially expressed pathways identified in **Supplementary Table 2** is displayed, representing ECM generation, cell-cell interactions, chemokine signaling, antigen presentation, interferon response, and cell division. Pathway enrichment scores were normalized across groups for comparison.

The 21, 060 fibroblasts contained within the 3 clusters and comprising all 4 sorted populations were isolated and reclustered, distinguishing 7 groups (**Figure 3A**). Groups G1 and G2 were significantly enriched for *Fap* (**Figure 3B**; **Supplementary Table 1**), a known indicator of pro-tumor function in CAF (15, 16), which we previously associated with CAF outside of TLS (11). Despite the evident presence of a large number of *Fap*-expressing cells in G0 and a higher mean level of *Fap* expression in these cells, there was not a statistical enrichment of overall *Fap* expression in G0 (**Figure 3B**; **Supplementary Table 1**). Conversely, groups G3, G4, G5, and G6 were significantly enriched for *Vcam1* (**Figure 3B**; **Supplementary Table 1**), which we previously associated with CAF inside TLS (11). Expression of *April* (*Tnfsf13*) and *Baff* (*Tnfsf13b*), both of which promote B cell survival and were expressed at much higher levels in CAF from TLS containing tumors (11), was modestly enriched in G0 and G5, and G1 and G2, respectively (**Supplementary Table 1**). Importantly, expression of *Cxcl13* was significantly and selectively enriched in G3, and while not reaching the threshold for significance, also expressed in a larger fraction of cells in G6 (**Figure 3B**; **Supplementary Table 1**). Thus, the characteristics of CAF that we previously associated with TLS development were differentially enriched in multiple CAF groups, but the data suggest that G3 is the primary driver of B cell accumulation based on its expression of *Cxcl13*.

This distinction in group functionality was also evident when CAF populations that had been sorted based on expression of tdTomato and GFP were identified on the UMAP plot. tdTomato^neg^ eGFP^neg^ cells were distributed among all groups, but were concentrated in G0 and G1, and to a lesser extent in G6 and G5 (**Figure 3C**). The distributions of the tdTomato^+^ eGFP^neg^ and tdTomato^+^ eGFP^+^ cells were almost identical, with roughly 50% in G3 and 22% in G6, and only small fractions distributed into the remaining groups. This is in keeping with the enrichment of *Cxcl13*^+^ cells in these same groups. Finally, the tdTomato^neg^ eGFP^+^ cells were distributed similarly into G3, G5, and G4, with a somewhat elevated fraction in G6. Because GFP expression is dependent on prior expression of Cxcl13-cre, these results indicate that at least some of the cells in G5 and G4 originate from cells in G3 and/or G6. Despite their differences in *Cxcl13* expression, the proximity of cells in groups G3-G6 in the UMAP plot also suggests broad similarities in their overall function.

To gain greater insight into the functional properties of the different CAF groups, we performed gene set enrichment analysis to identify the most differentially expressed and immunologically relevant gene sets (**Supplementary Table 1**). **Figure 3D** shows a representative subset of functionally relevant pathways selected based on statistically significant and positive enrichment among individual groups. Cells in G1 and G2, and to a lesser extent G3, were highly enriched for ECM pathways, including those focused on components of collagen, basement membrane, and interstitial matrix. Conversely, cells in G3, G4, and G6 were enriched in pathways involving heterotypic cell-cell adhesion, cell-cell contact, and junction formation. Cells in all groups except G0 and G5 were enriched in terms related to chemokine production and activity, and regulation of leukocyte migration. Interestingly, while cells in G1 and G2 were particularly enriched for Cxcl12 activity, cells in G3 and to a lesser extent G6 and G4, were specifically enriched for B cell chemotaxis, consistent with their expression of *Cxcl13*. Cells in G5, G3, G6, and to a lesser extent G4, were highly enriched for MHC-II and MHC-I antigen presentation pathways, and similarly, responses to Type I and II interferons. Finally, cells in G2 and G6 were particularly enriched in terms associated with cell proliferation and division. Importantly, these terms are in large part concerned with modulation of immunity, and with cell trafficking and localization. The individual enriched genes that underlie these functions are described in more detail below.

### 2.4 Group G0 represents protoCAF

**Figure 4:**
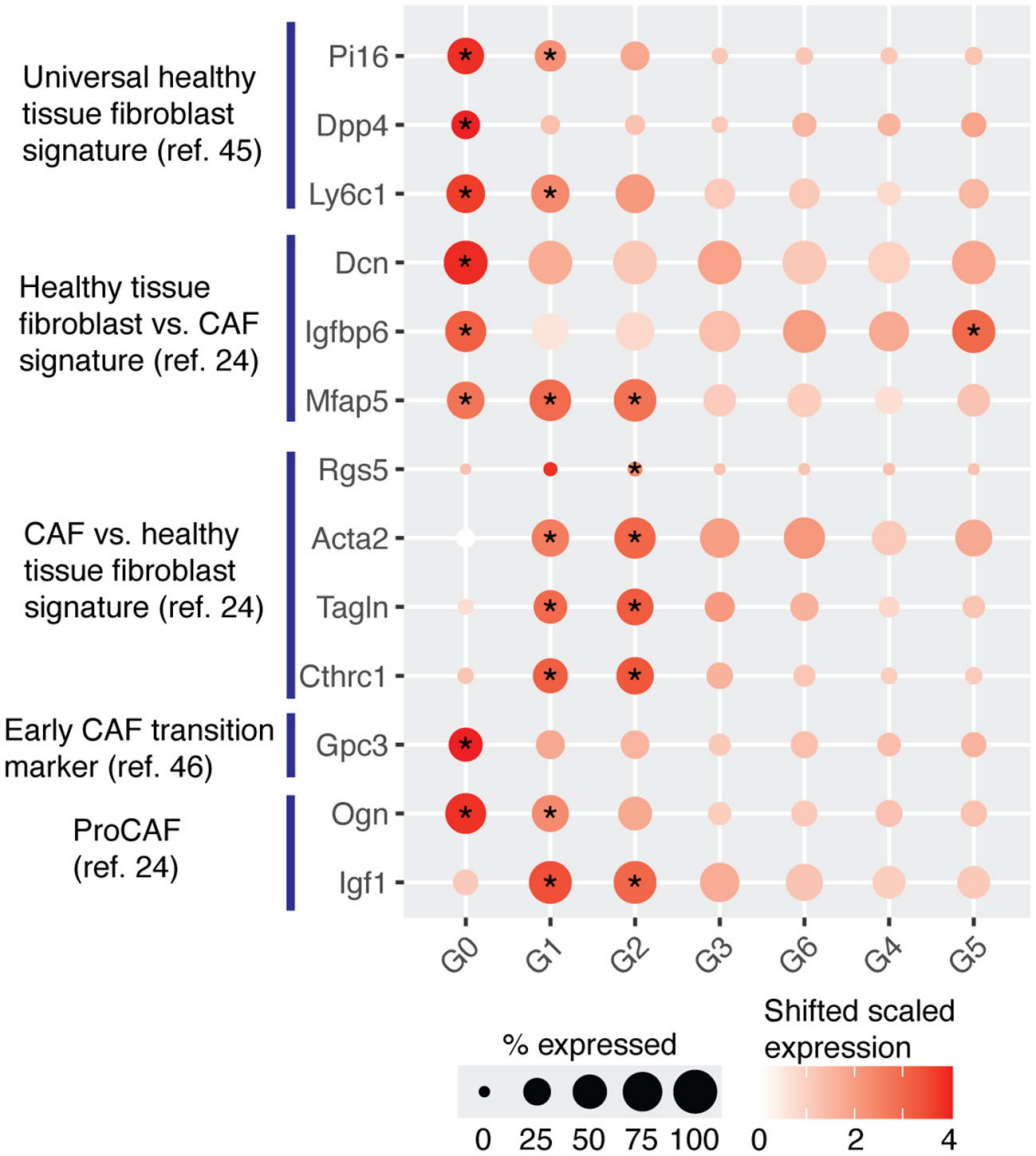
Enriched genes in G0 define protoCAF characteristics. Genes defining terms associated with fibroblast and CAF development were chosen based on the indicated references. Gene expression was normalized across groups and shifted to establish a baseline of 0. Dot size indicates percentage of cells positive for the gene. Color intensity indicates normalized expression intensity. A Wilcoxon Ranked Sum test was run to identify genes significantly enriched in each group. Those marked for significance (*) had a Bonferonni-Hochburg adjusted p value < 0.05, a positive log2 fold change > 0.25, and were expressed in > 10% of cells in the group in question. Data supporting this determination are shown in **Supplementary Table 3**.

In contrast to the groups described above, the G0 group did not display enrichment for any immunologically relevant pathways, nor those associated with specialized fibroblast functions (**Figure 3D**; **Supplementary Table 2**). Instead, the pathway terms enriched in G0 reflected functions associated with cell polarity and the cytoskeleton (**Supplementary Table 2**). To gain more insight into this group, we turned to analysis of the most differentially expressed individual genes. Among the most differentially expressed genes in Group G0 were *Pi16, Dpp4, and Ly6c1* (**Figure 4**; **Supplementary Table 3**). These genes have been observed to identify a ‘universal’ healthy tissue fibroblast population that has not yet specialized in function (45). An additional set of differentially expressed genes in this group included *Dcn, Igfbp6, and Mfap5*. These were enriched in healthy tissue fibroblasts compared to CAF from the same tissue (24). In this same study (24), *Rgs5, Acta2, Tagln*, and *Cthrc1* were under-expressed in healthy tissue fibroblasts compared with CAF. We found that *Rgs5* was minimally expressed in any of our fibroblasts, while expression of the remaining genes, which are involved in either the actin cytoskeleton or ECM remodeling, was substantially lower in G0 than in many or all other groups (**Table 1**). Conversely, G0 CAF were enriched for expression of *Gpc3*, a gene enriched in CAF inferred to be derived from early-stage *Pi16*^+^ precursors (46). Finally, G0 CAF were enriched for expression of *Ogn*, one of 3 markers of progenitor CAF defined by Chen et al. (24), while *Igf1*, another progenitor marker, was enriched in G1 and G2, and a 3^rd^, *C7*, was not significantly expressed by any group (**Figure 4**; **Supplementary Table 3**). Collectively, the overexpression of a combination of healthy tissue fibroblast genes and early-stage CAF genes suggest that group G0 comprises ‘protoCAF’ that have recently transitioned from normal fibroblasts.

**Table 1:** Relative expression of normal and cancer associated fibroblast enriched genes in group G0.

Gene	avg_log2FC	pct.1	pct.2	p_val_adj
*Pi16*	4.7	0.601	0.111	0
*Gpc3*	3.52	0.484	0.199	0
*Ly6c1*	3.2	0.707	0.403	0
*Dcn*	2.12	0.994	0.97	0
*Igfbp6*	1.33	0.836	0.818	5.45E-221
*Dpp4*	3.7	0.285	0.102	4.07E-170
*Mfap5*	1.44	0.648	0.524	2.48E-142
*Rgs5*	-5.62	0	0.003	1
*Cthrc1*	-2.46	0.025	0.221	7.79E-95
*Tagln*	-4.47	0.019	0.292	2.51E-153
*Acta2*	-3.7	0.057	0.706	0

A Wilcoxon ranked-sum test was used to compare expression of the indicated genes among CAF groups. *Avg_log2FC*, log2 fold change of gene expression by cells in the group of interest relative to cells outside the group of interest. *Pct.1*, percentage of expressing cells in the group of interest. *Pct.2*, percentage of expressing cells outside the group of interest. *P_val_adj*, p value adjusted using Bonferroni-Hochberg correction.

### 2.5 Fibroblast groups are polarized between ECM deposition and direct cell-cell interaction

**Figure 5:**
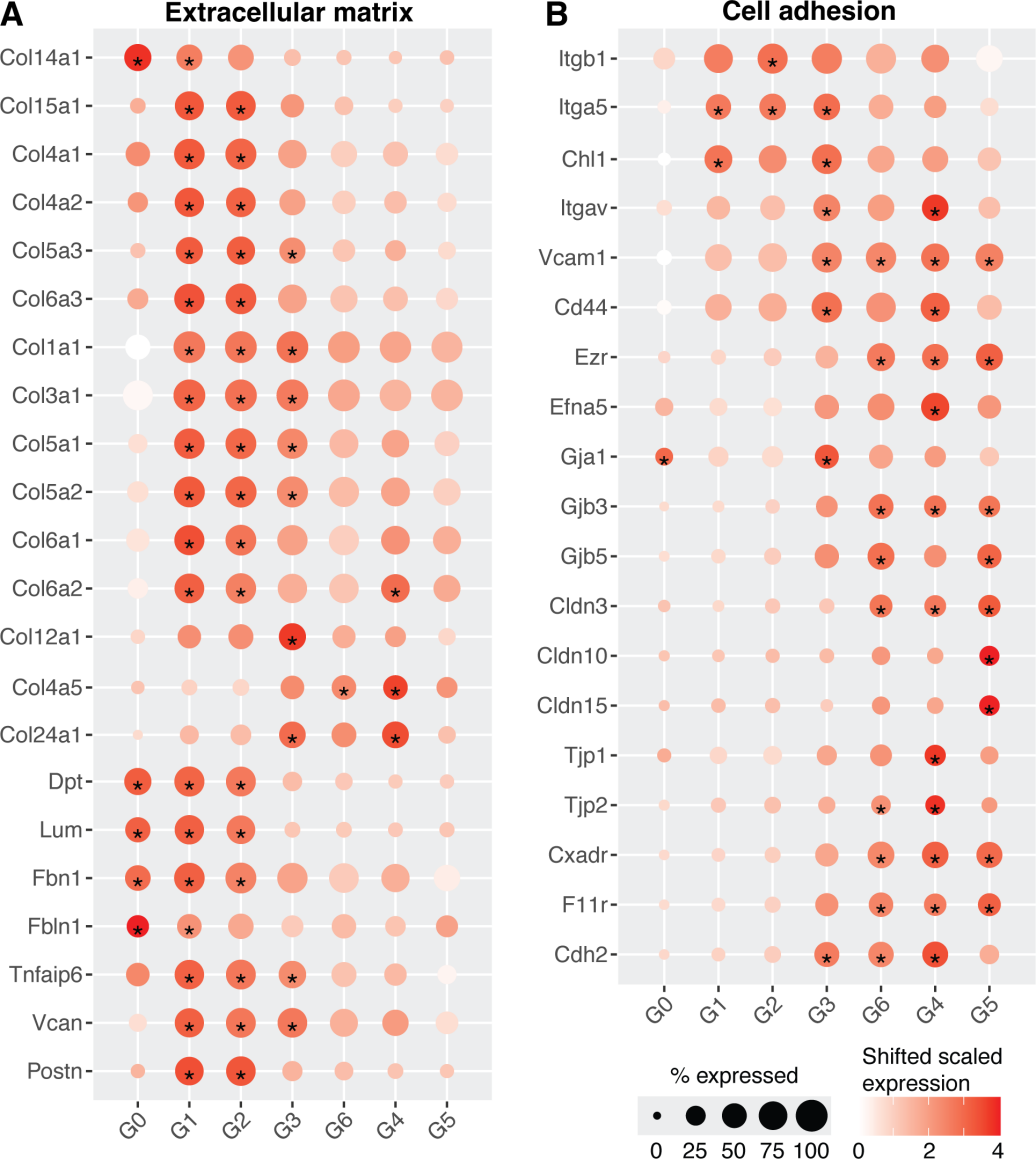
Distribution of genes associated with **(A)** ECM deposition and remodeling and **(B)** cell adhesion among CAF groups. For each gene, mean expression values for each group were normalized and shifted to establish a baseline of 0. Color intensity indicates normalized expression intensity. Dot size indicates percentage of cells positive for the gene by group. A Wilcoxon Ranked Sum test was run to identify genes significantly enriched in each group. Those marked for significance (*) had a Bonferonni-Hochburg adjusted p value < 0.05, a positive log2 fold change > 0.25, and were expressed in > 10% of cells in the group in question Data supporting this determination are shown in **Supplementary Table 3**.

ECM deposition by CAF contributes to tumor growth by preventing immune cell trafficking into the tumor (47). While G0 was selectively enriched only for *Col14a1*, groups G1 and G2 were strongly enriched for genes encoding a wide range of fibrillar, microfibril-associated, and basement membrane-associated collagens (**Figure 5A**; **Supplementary Table 3**). Groups G0, G1, and G2 were also strongly enriched for genes encoding proteins involved in fibrillogenesis (*Dpt, Lum*) and microfibril formation (*Fbn1, Fbln1*). G1 and G2 were additionally enriched for genes encoding proteins that mediate incorporation of hyaluronic acid (HA) into ECM structures (*Tnfaip6, Vcan*), and periostin (*Postn*), which encodes a secreted protein identified as a part of the ECM, and which serves as a ligand for αv integrins (48, 49). Group G3 was enriched for some of these collagen and ECM generation-related genes, but expression relative to G1 and G2 was usually noticeably decreased. Further reductions in the expression of these genes were evident in G6, G4, and G5. However, G3, G6, and G4 were selectively enriched for *Col12a1*, *Col4a5*, and/or *Col24a1*. These patterns in gene enrichment suggest groups G1 and G2 play a large role in constructing and modifying the ECM, while groups G0 and G3–G6 play less significant and/or more specialized roles.

There was also a distinction in group expression of genes encoding molecules involved in cell adhesion (**Figure 5B**; **Supplementary Table 3**). *Itga5* and *Itgb1*, which form α5β1 integrin, and *Chl1*, were enriched in G1, G2, and/or G3, while *Itgav* was enriched in G3 and G6. CHL1 has multiple ligands and is involved in regulation of many cell processes, but the primary ligand for α5βa1 integrin is fibronectin, a component of the ECM (50, 51). The integrin αv chain pairs with 5 different β chains, and each of these integrins binds to a larger range of ligands than α5β1 integrin, but most of these ligands are also elements of the ECM (50, 51). The differential expression of these molecules is consistent with the engagement of these groups with different components of the ECM, suggesting differences in their localization. In addition, CAF in group G4, and to a lesser extent G3, G6, and G5, were enriched for expression of genes encoding proteins involved in cell-cell interactions, including *Vcam1*, *Cd44*, *Ezr*, and *Efna5*. VCAM1 is a primary ligand for α4β1 integrin, which is widely expressed on activated lymphoid and myeloid cells and mediates cell-cell adhesion and extravasation (52, 53). CD44 binds strongly to HA, enabling interaction with ECM (54). However, CD44^+^ fibroblasts have also been shown to coat themselves with HA, enabling them to bind to CD44^+^ monocytes (55). EZRIN (*Ezr*), a cytoplasmic protein linking the actin cytoskeleton to different cell surface molecules, has been shown to engage with both VCAM1 and CD44 (56, 57). EFNA5 is a ligand for ephrin A receptors, and its engagement can lead to bi-directional regulation of cell-cell adhesion, morphology, and proliferation (58). CAF in groups G6, G4, and G5, and to a lesser extent G3, were also enriched for expression of genes encoding proteins that mediate formation of different cell-cell junctions (**Figure 5B**; **Supplementary Table 3**). These include the gap junction proteins GJA1, GJB3, AND GJB5; several proteins that mediate formation of tight junctions (CLDN3, CLDN10, CLDN15, TJP1, TJP2, CXADR, F11R); and N-cadherin (*Cdh2*), which is involved in some adherens junctions (59). The enriched expression of these genes suggests that these groups are more involved in the formation of direct connections with one another and potentially with immune cells, than are cells in groups G0, G1, and G2.

### 2.6 Fibroblast groups are polarized to chemotactically attract different immune cell populations

**Figure 6:**
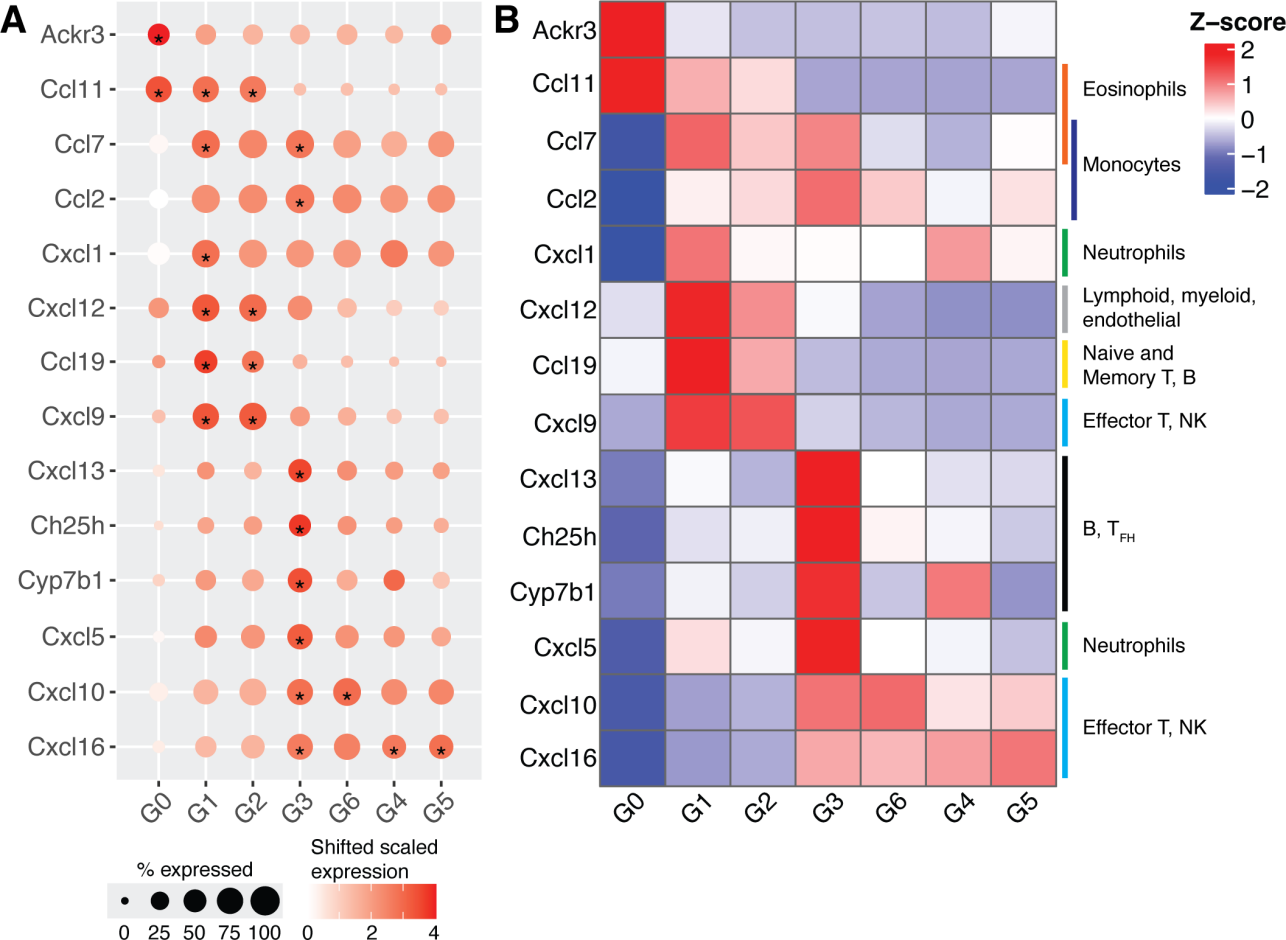
Distribution of genes associated with immune cell recruitment among CAF groups. **(A)** Expression of chemokines and chemokine-relevant modifiers. For each gene, mean expression values for each group were normalized and shifted to establish a baseline of 0. Color intensity indicates normalized expression intensity. Dot size indicates percentage of cells positive for the gene by group. A Wilcoxon Ranked Sum test was run to identify genes significantly enriched in each group. Those marked for significance (*) had a Bonferonni-Hochburg adjusted p value < 0.05, a positive log2 fold change > 0.25, and were expressed in > 10% of cells in the group in question Data supporting this determination are shown in **Supplementary Table 3**, **Supplementary Figure 3**. **(B)** Normalized gene expression data from **(A)** was replotted in a heatmap to better visualize differences in expression between groups. Labels on the right side of the heatmap denote target leukocytes responding to each chemokine.

We previously showed that, in addition to CXCL13 and its receptor, CXCR5, TLS formation in tumors is also dependent on the chemokine CCL21 and its receptor, CCR7, and the primary source of both chemokines was CAF (9, 11). Surprisingly, we detected only low level *Ccl21* expression in only 13 cells, with no concentration in any group. Given our prior robust detection of Ccl21 by immunofluorescence and RT-PCR (9, 11), we believe this to be a technical issue in the scRNA-seq approach. However, we did detect *Ccl19* (**Supplementary Table 3**), which also binds to CCR7 and is also differentially upregulated in tumors containing TLS (9). This led us to consider what additional chemokines were made by different CAF groups. All groups expressed multiple genes encoding different chemokines, but only a single chemokine receptor, *Ackr3*, which was differentially expressed by G0 (**Figure 6**; **Supplementary Figure 3**, **Supplementary Table 3**). ACKR3 is an unusual chemokine receptor that scavenges CXCL12 and CXCL11 and is thought to function as a decoy, but also mediates adhesion, migration, and survival (60–63). G0, G1, and G2 were enriched for expression of *Ccl11*, which encodes an eosinophil chemoattractant that has also been implicated as an endothelial cell angiogenic factor (64). G0 was not enriched for expression of any other chemokine. Conversely, G1 and/or G3 were enriched for expression of *Ccl7, Ccl2*, and *Cxcl1*, which encode chemokines that recruit eosinophils, monocytes, and neutrophils, respectively. Groups G1 and G2 also strongly and differentially expressed *Cxcl12, Ccl19*, and *Cxcl9*. CXCL12 attracts, retains, and activates a variety of CXCR4-expressing immune and non-immune cells, including some cancer cells (65–68). While CCL19 recruits CCR7-expressing naïve T and B cells and central memory T cells into secondary lymphoid tissue, CXCL9 recruits CXCR3-expressing T effector and NK cells from bloodstream into inflamed tissue, and we previously showed the importance of this pathway in this murine melanoma model (69). Thus, cells in G1 and G2 appear poised to recruit both naïve and effector lymphocytes into tumors from the bloodstream, promoting an essential preliminary step in TLS development.

As shown earlier, group G3 strongly and differentially expressed *Cxcl13*. G3 was also enriched for expression of *Ch25h* and *Cyp7b1* (**Figure 6**; **Supplementary Figure 3**; **Supplementary Table 3**), which encode proteins that convert cholesterol to 7-alpha, 25-dihydroxycholesterol. This molecule is a ligand for GPR183, another G-protein coupled receptor that regulates B and T cell migration to germinal centers (70). Given this, it was surprising that G3 was also enriched for expression of *Cxcl5*, which encodes a neutrophil attractant. Lastly, G3 and G6 were enriched for expression of *Cxcl10*, which like *Cxcl9*, encodes a chemokine that binds to CXCR3 to recruit effector T and NK cells (71), while G3-G6 were enriched for expression of *Cxcl16*, which binds to CXCR6^+^ T and NK cells. CXCL16 exists in both soluble and membrane-bound forms (72), and its membrane bound form has been shown to anchor T cells to CXCL16-expressing fibroblasts (73) suggesting it may function as an organizer as well as a recruiter. Collectively, these results suggest that all CAF groups can recruit one or more myeloid populations and thereby the potential to either enhance or diminish anti-tumor immunity and tumor growth. However, groups G1, G2, and G3 also act distinctly to either recruit B and T lymphocytes of different activation states into tumors, or to organize them into TLS, while groups G0, G4, G5, and G6 play less important or negligible roles.

### 2.7 Select CAF groups are enriched for expression of multiple genes involved in antigen presentation

**Figure 7:**
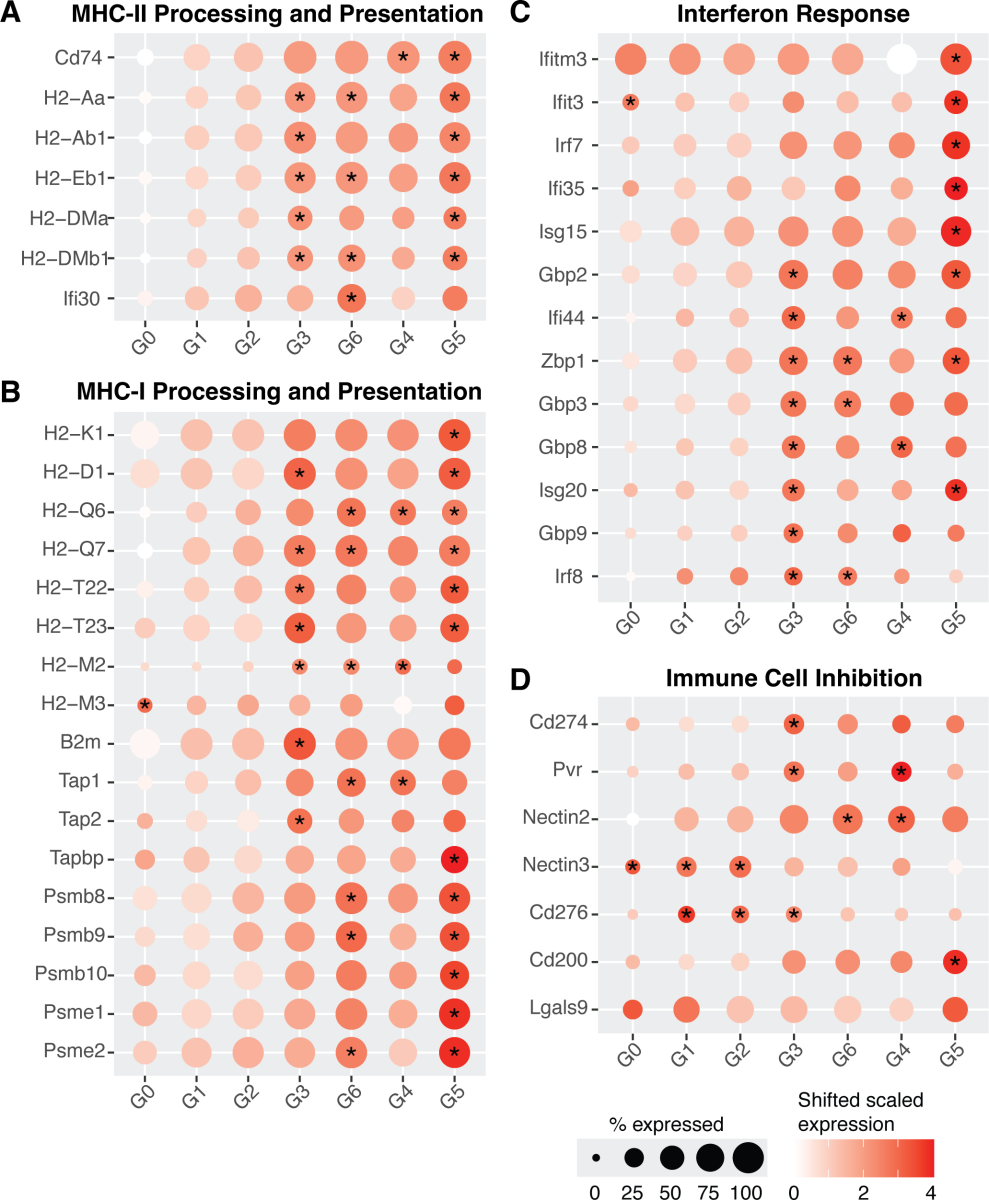
Expression of genes associated with MHC-restricted antigen presentation and interferon response pathways in CAF groups. Expression of genes involved in **(A)** MHC-II antigen presentation, **(B)** MHC-I antigen presentation, **(C)** Interferon response, and **(D)** immune cell inhibition are displayed. For each gene, mean expression values for each group were normalized and shifted to establish a baseline of 0. Color intensity indicates normalized expression intensity. Dot size indicates percentage of cells positive for the gene by group. A Wilcoxon Ranked Sum test was run to identify genes significantly enriched in each group. Those marked for significance (*) had a Bonferonni-Hochburg adjusted p value < 0.05, a positive log2 fold change > 0.25, and were expressed in > 10% of cells in the group in question Data supporting this determination are shown in **Supplementary Table 3**, **Supplementary Figure 4**.

Group G5, and to a lesser extent G3 and G6, but not G0, G1, or G2, were enriched for expression of all or some MHC-II structural genes (*CD74, H2-Aa1, H2-Ab1, H2-Eb1*), and components of the MHC-II processing and presentation pathway (*H2-DMa, H2-DMb, Ifi30*) (**Figure 7A**; **Supplementary Table 3**). Similarly, they were enriched for expression of multiple classical MHC-I structural genes (*H2-K1, H2-D1, B2m*), non-classical MHC-I structural genes (*H2-Q6, H2-Q7, H2-T22, H2-T23, H2-M2, H2-M3*), as well as genes encoding both subunits of the TAP peptide transporter, the TAP binding protein (*Tapbp*), and 5 components of the immunoproteasome (*Psmb8, Psmb9, Psmb10, Psme1*, and *Psme2*) (**Figure 7B**; **Supplementary Table 3**). These same groups were also enriched for expression of several other genes that are transcriptionally upregulated by Type I IFN and IFNγ signaling (**Figure 7C**; **Supplementary Table 3**), suggesting that the enrichment of MHC-II and -I pathway gene expression may also be in response to these signals.

Interestingly, however, there was no evident expression in any CAF group of genes encoding costimulatory ligands that could lead to effective T cell activation and immunity (*Cd80, Cd86, Tnfsf4 (Ox40L), Tnfsf5 (Cd40L), Tnfsf7 (Cd70), Tnfsf8 (Cd30L), Tnfsf9 (4-1BBL), GitrL (Tnfsf18))* (**Supplementary Figure 4**; **Supplementary Table 3**). Conversely, groups G3, G6, and or G4 showed variably enriched expression of *Cd274* (PD-L1), *Pvr* (CD155), and/or *nectin2* (CD112), which encode co-inhibitory molecules that ligate PD1, TIGIT, and CD112R, respectively (**Figure 7D**; **Supplementary Figure 4**; **Supplementary Table 3**) (74, 75). The elevated expression of MHC-II genes in groups G5, G3, and G6 also creates the possibility of co-inhibition via LAG3. G1 and G2 were enriched for expression of *nectin3* (CD113) and *Cd276* (B7-H3), which encode alternate co-inhibitory molecules that bind to TIGIT and an unidentified receptor, respectively (74, 75), and G5 was enriched for expression of *CD200*, which encodes a protein that acts as broad-based inhibitor of immunity (74). All groups show significant expression of *Lgals9* (galectin 9), which encodes a ligand for TIM3, although no group showed enriched expression. Overall, these results suggest the possibility that different CAF groups can regulate intratumoral immunity via different pathways, but this also depends on the ability of these groups to present relevant antigens via either MHC-II or MHC-I, which we have not addressed.

### 2.8 Cytokine and cytokine receptor expression varies widely among CAF groups

**Figure 8:**
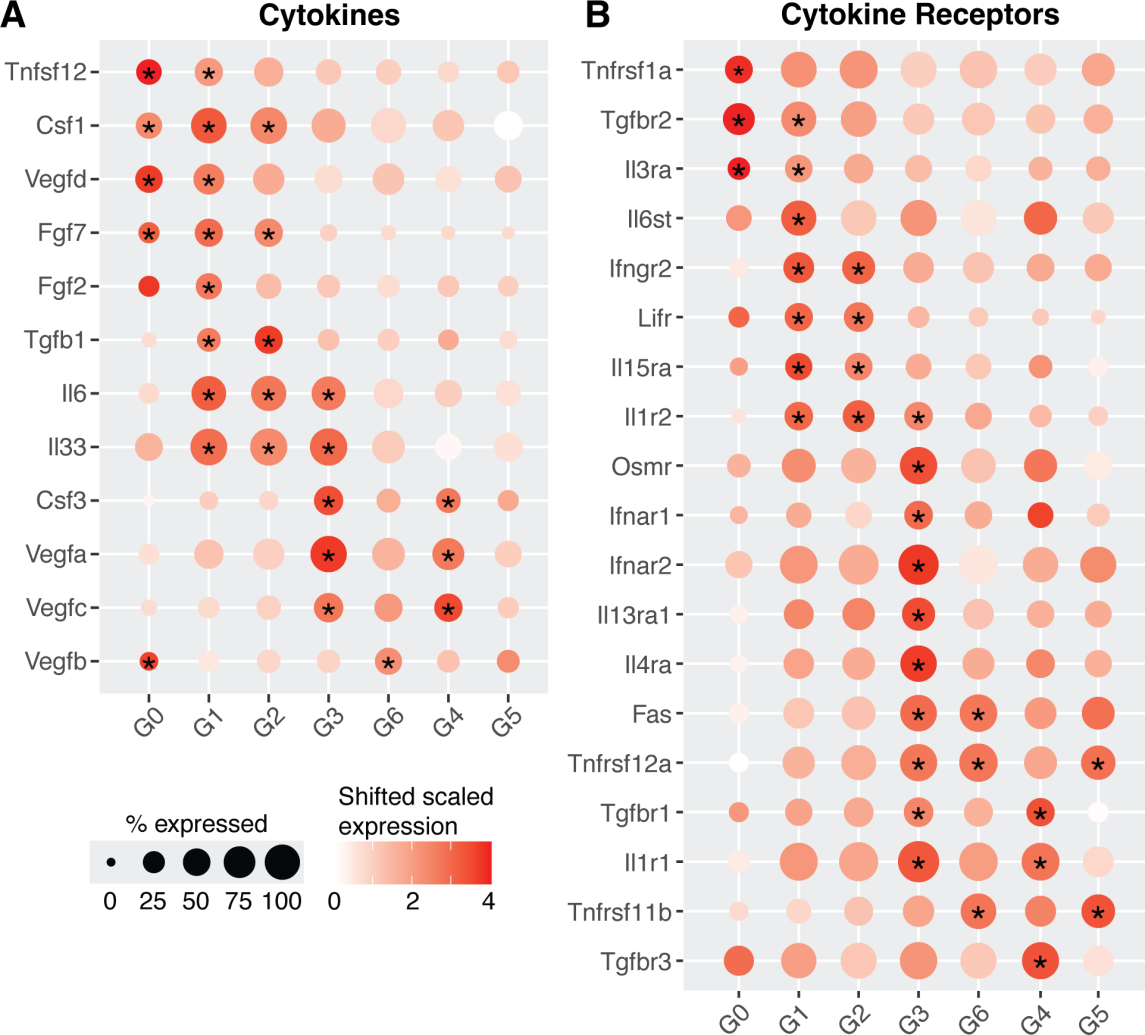
Expression of cytokines and receptors among CAF groups. Expression of **(A)** cytokines and **(B)** cytokine receptors amongst CAF groups. For each gene, mean expression values for each group were normalized and shifted to establish a baseline of 0. Color intensity indicates normalized expression intensity. Dot size indicates percentage of cells positive for the gene by group. A Wilcoxon Ranked Sum test was run to identify genes significantly enriched in each group. Those marked for significance (*) had a Bonferonni-Hochburg adjusted p value < 0.05, a positive log2 fold change > 0.25, and were expressed in > 10% of cells in the group in question Data supporting this determination are shown in **Supplementary Table 3**, **Supplementary Figures 5**, **6**.

Expression of genes encoding most immune-relevant cytokines, including interferons, interleukins, CSF and TGF family members, and non-co-stimulatory TNF superfamily members, was limited and variable in each CAF group, and overall (**Supplementary Figure 5**). Groups G0 and G1 were enriched for expression of *Tnfsf12* (TWEAK) and *Vegfd*, while G0, G1, and G2 were enriched for expression of *Csf1* and *Fgf7*, and G1 was enriched for expression of *Fgf2* (**Figure 8A**; **Supplementary Figure 5**). TWEAK and FGF7 are involved in tissue injury responses (76, 77), while VEGFD is pro-angiogenic (78), and CSF1 promotes development of macrophages (79). Groups G1 and G2 were enriched for expression of *Tgfb1*, while G1, G2, and G3 were enriched for *Il6* and *Il33.* TGF-β has strong immunoregulatory activities (80). IL6 can promote a myofibroblastic phenotype and increase ECM deposition (81, 82), and polarize macrophages toward an M2 phenotype (83). Similarly, IL33 produced by CAF has been shown to drive immune responses that can either enhance or diminish tumor control (84–87). Finally, G3 and G4, were enriched for expression of *Csf3, Vegfa, and Vegfc*, while G6 and G1 were selectively enriched for expression of *Vegfb*. These results suggests that the well-established pro-tumor activities of CAF, many of which are concerned with angiogenesis and wound-healing immune responses, are distributed broadly but differentially through all the groups that we have identified.

Similarly, individual CAF groups expressed a limited number of receptors for immune-relevant cytokines, but a diverse collection overall (**Supplementary Figure 6**). G0 and G1 were enriched for expression of *Tnfrsf1a, Tgfbr2, Il3ra*, and/or *Il6st*, while G1 and G2 were enriched for expression of *Ifngr2, Lifr, Il15ra*, and *Il1r2* (**Figure 8B**; **Supplementary Figure 6**). G6 was enriched for *Fas, Tnfrsf12a* and *Tnfrsf11b*, G4 was enriched for *Tgfbr1*, *Il1r1*, and *Tgfbr3*, and G5 was enriched for expression of *Tnfrsf12a* and *Tnfrsf11b.* While the sensitivities of these groups are limited, they typically include both pro- and anti-inflammatory cytokines, raising interesting questions about how cells in these groups respond to their local environment. Strikingly, G3 was enriched for expression of a broad collection of cytokine receptors (*Il1r2*, *Osmr, Ifnar1, Ifnar2, Il13ra1, Il4ra, Fas, Tnfrsf12a, Tgfbr1*, and *Il1r1*). Thus, CAF in group G3 appear to be sensitive to a wide range of cytokine signals associated with innate, Th1, Th2, and Treg responses.

## 3 Discussion

In this study, we used a mouse model in which TLS develop spontaneously in IP B16 melanoma tumors to identify the mechanisms that promote TLS development, with a particular focus on CAF subpopulations, and molecules that have been implicated in TLS development in work done by us and by other groups. We extended earlier work that demonstrated that CXCR5-expressing B cells are essential for TLS development (11), and established that CXCL13, the ligand for CXCR5, is expressed exclusively by a population of CAF, and that these CAF are essential to promote B cell accumulation in these tumors. In keeping with our earlier work showing that the presence of TLS was dependent on B cells, and associated with both smaller tumor size and response to checkpoint blockade therapy (11), we also found that the lack of B cell accumulation in the absence of Cxcl13^+^ CAF was associated with larger tumor size. However, the absence of Cxcl13^+^ CAF also was associated with alterations in the number of macrophages, and of Cxcl13^neg^ CAF. This points to complexity in the relationships between these CAF populations, such as providing supportive growth factors or intratumoral niches, as well as the loss of a successor (tdTomato^neg^ eGFP^+^) population. Consequently, enhanced tumor outgrowth might also reflect alterations in the balance of tumor-promoting and tumor-suppressing CAF and/or macrophages. Using scRNA-seq, we identified 7 subpopulations of CAF in TLS containing tumors. While some of these CAF groups appear similar to those defined in human cancers and other murine tumor models, others appear novel, and one subpopulation was substantially enriched for CXCL13-expressing cells. This population was also enriched for additional genes that could promote B cell recruitment and enable regulation by a variety of cytokines. However, other genes that we have shown or hypothesize to be important in TLS development showed enriched expression in other CAF groups. Thus, TLS development in this model appears to depend on an unusual CAF population secreting CXCL13, together with additional CAF populations that operate at different points in the process.

In the IP tumor model evaluated in this study, the sole source of CXCL13 was CAF. There are numerous studies in other tumor models that point to alternative sources of CXCL13 that may contribute to TLS formation, including PD-1^hi^ CD8 T cells (28–30, 35), CD4 T cells (31–35), endothelial cells (34), as well as CAF with a follicular dendritic cell-like phenotype (31, 35, 36). While most of these studies point to a positive impact of these different cell sources on TLS formation, maturation, and/or tumor outgrowth and patient survival, there are exceptions (88, 89). One study suggested that early TLS were associated with CXCL13^+^ CD4 T cells, while more mature follicle containing TLS were associated with CXCL13^+^ CAF (31), but there is otherwise little information to suggest how CXCL13 expression by these different populations impacts either TLS development or tumor growth. This is an important question for future research.

Over the last several years, many reports using scRNA-seq have documented the presence of multiple subpopulations of CAF in different human cancers and mouse tumor models. Pan-cancer analyses and recent reviews have established a consensus identification of 4 main CAF groups: progenitor (proCAF), myofibroblastic or matrix producing (myCAF or mCAF), inflammatory (iCAF), and antigen-presenting (apCAF) (23–27, 90–92). Studies in some tumor types have identified additional subpopulations (25–27), of which vascular (vCAF), interferon-responsive (ifnCAF), reticular (rCAF), and proliferating (pCAF) are most significant to our work. These latter subpopulations are usually subsets of the main CAF groups. More recently, this has been complemented by single cell spatial multi-omics (93), which led to the identification of 4 CAF subtypes in both lung adenocarcinoma and head and neck cancer. While these subtypes did not correspond precisely to the main CAF groups described above, the analysis suggested that myCAF/mCAF-like cells localized in tumor adjacent and stromal regions, while iCAF-like and ap/r-like CAF localized to myeloid and lymphoid aggregate/TLS regions, respectively. Taken together, these results establish that CAF are comprised of multiple populations with distinct functions that are attuned to and/or dependent on their intratumoral location.

We have attempted to classify the 7 populations that we identified in terms of these earlier defined CAF subpopulations using a consensus of apparently uniquely expressed genes (**Supplementary Figure 7**; **Supplementary Tables 4**, **5**). Based on enriched expression of ≥50% of population-defining genes, groups G1 and G2 both typify the mCAF and Spatial S1/S2 population, and G1 also typifies myCAF (**Supplementary Tables 4**, **5**). However, G1 and G2 are also both enriched for *Ccl19* and *Cxcl9*, hallmarks of rCAF and ifnCAF, respectively, and G2 also expresses several cell cycle/proliferating genes, a hallmark of pCAF. Our data suggest that the G0 group represents proCAF, but it also is enriched for expression of several genes that are considered signatures for iCAF (*Fbln1, Dpt, Igfbp6, Csf1, C4b, Cd34, Gsn*), and it does not express any genes that would suggest a spatial localization (**Supplementary Tables 4**, **5**). Similarly, based on selectively enriched expression of MHC-II associated genes, groups G5 and G3 typify apCAF, and G3 is associated with the spatial S3 and S4 populations (**Supplementary Tables 4**, **5**). Their overall upregulation of multiple IFN signaling pathway target genes might also suggest identifying them as ifnCAF, although this category has not been defined in this way, and has also been defined in some but not all studies to exclude upregulation of MHC-II genes (46, 94, 95). Groups G6 and G4 are the most difficult to assign as they are only enriched for 2–5 of the CAF population defining genes overall (**Supplementary Tables 4**, **5**). While they appear the most closely aligned to apCAF, the number of genes that define this category is overall quite small. Thus, while many of the CAF groups that we identified correspond loosely to the consensus subpopulations defined in other studies, there is also a considerable degree of ambiguity. Some of this ambiguity is likely to be a consequence of the specific tumor and tissue microenvironment in which the CAF reside, as suggested by similar variations in CAF populations identified in different human tumor types (23, 25–27). Prior work in B16 melanoma (96) identified 3 CAF subpopulations: S1 immune, S2 desmoplastic, and S3 contractile. The S1 population most closely resembled iCAF, and S2 resembled myCAF/mCAF, while S3 shared characteristics with vCAF, but also with pericytes. Similar populations were also identified in human melanoma (97). However, a comparison of chemokine/cytokine and ECM gene expression patterns establishes that the G0, G1, and G2 groups defined here overlap strongly with both the S1 and S2 subpopulations. Thus, CAF groups in IP tumors differ from their SC counterparts in part by gene expression patterns that blur SC CAF subpopulation distinctions. However, they also differ in that IP tumors contain at least 4 additional CAF populations. These additional groups (G3, G4, G5, G6) lie in close proximity on the UMAP projection, pointing to their overall similarity to one another and distinctiveness from the other groups. We suggest that the development of TLS in the B16 melanoma IP tumors studied here is accompanied by, and at least partially dependent on, the differentiation of these 4 additional CAF populations. In addition, based on their expression of eGFP in the absence of tdTomato, many of the cells in G4 and G5 expressed CXCL13 at an earlier point, leading to activation of eGFP expression, but no longer did so at the time of tumor harvest. This indicates that these cells have arisen in part from cells in G3 and/or G6. To our knowledge, despite many prior applications of pseudotime analyses to CAF populations to infer relationships, this is a novel finding for CAF.

An alternate approach to the classification issue is to identify the genes that are selectively enriched only in a single group, which thereby may serve as group-selective markers as well as illuminating group functionality (**Table 2**). From this perspective, G0 not only selectively expresses proCAF markers, but also *Tnfsf1a, H2-*M3, and *Lgals9* (a ligand for Tim3), pointing to unique responsiveness to a cytokine as well as antigen presentation capability. G1 and G2 individually express very few genes selectively, but collectively, they selectively express multiple ECM, myofibroblastic, and cell adhesion molecules, as well as chemokines, cytokines, and cytokine receptors (**Table 2**). This suggests that these cells function to create scaffolding and structure, but also to selectively recruit naïve and effector lymphocytes (*Cxcl12, Ccl19, Cxcl9*), to receive pro-immune signals (*Ifngr2, IL15ra*) and deliver pro-tumor immunoregulatory stimuli (*Igf1, Tgfb1*). In this sense, they correspond to a composite of mCAF, myCAF, and rCAF. Conversely, G5 selectively expresses two claudin genes, a myosin light chain isoform, and several genes associated with MHC-I antigen presentation and interferon responsiveness. In this sense, they correspond to a composite of apCAF and ifnCAF. Although very small fractions of G3 selectively express Mmp isoforms and a myosin heavy chain isoform (**Supplementary Figure 7**), they strongly selectively express chemokines associated with recruitment of B cells, monocytes, and neutrophils (*Cxcl13, Ch25h, Cyp7b1, Ccl2, Cxcl5)*; pro-inflammatory cytokine receptors (*Ifnar1, Ifnar2, Osmr, Il13ra1*); MHC-I antigen presentation (*B2m, Tap2*); and immune inhibition (*Gbp9, Cd274*)m (**Table 2**), pointing to a rich functionality that does not easily fit within previously delineated populations. Once again, G6 and G4 are difficult to classify in this analysis. While these groups selectively express 3–4 genes, they provide little confidence in assigning an overall function.

**Table 2:** Key marker genes to discriminate G0 through G6 CAF groups.

Group	Fibroblast lineage	ECM/Cell adhesion	Myofibroblast	Chemokines/receptors	Cytokines/receptors	Antigen Presentation/interferon response
G0	*Dpp4, Gpc3*	*Dcn*		*Ackr3*	*Tnfrsf1a*	*H-2M3, Lgals9*
G1		*Mmp11*		*Cxcl1*	*Fgf2, Il6st*	
G2		*Itgb1*				
G1+G2		*Collagens 15a1, 4a1, 4a2, 6a3, 6a1, Cthrc1, Postn, Mmp14*	*Acta2, Tagln, Myh9, Myl9*	*Cxcl12, Ccl19, Cxcl9*	*Ifngr2, IL15ra, Lifr, Igf1, Tgfb1*	
G3		*Mmp8, 9, 10, 19, 27*	*Myh11*	*Cxcl13, Ch25h, Cyp7b1, Ccl2, Cxcl5*	*Ifnar1, Ifnar2, Osmr, Il13ra1, Lif*	*B2m, Tap2, Gbp9, Cd274*
G6		*Mmp17*	*Myh10, Mylpf*			*Ifi30*
G4		*Efna5, Tjp1*			*Tgfbr3*	
G5		*Cldn10, Cldn15*	*Myl12b*			*H2-K1, Tapbp, Psmb10, Psme1, Ifitm3, Irf7, Ifi35, Isg15, Cd200*

Genes were identified from data in **Figures 4**–**8**, **Supplementary Figure 7** based on statistically significant enhanced expression in scRNA-seq data only in the associated group.

It is also possible to infer functionality without the demand for selective gene expression by a single group, and by comparing similar groups. As a primary example, G4 is enriched for genes encoding a small subset of collagens, several cell adhesion molecules, *Cxcl16, Cd74, Vegfa, Vegfc*, and receptors for TGFβ, IL1, and RANKL. While pointing to significant functional diversity, most of these genes have not been clearly associated with a major CAF population. As a second example, as cited above, G1 and G2 are functionally very similar, but G2 is enriched relative to G1 for genes associated with proliferation (Mki67, Top2a, and Cdk1) (**Supplementary Table 3**), suggesting it has characteristics of pCAF. As a third example, while nominally CXCL13^+^ (tdTomato^+^) CAF preferentially distribute into groups G3 and G6, these two groups are distinct based on expression of other genes. G6, like G2, is enriched relative to G3 for the same cell proliferation associated genes. G6 is also enriched relative to G3 for genes encoding cell-cell junction molecules, a distinct set of MHC-I pathway molecules, and VEGFB. However, like G4, this pattern of enriched gene expression still does not confer a strong sense of a CAF subpopulation with a refined function. Some of this uncertainty may arise from the fact that both tdTomato^neg^ eGFP^neg^ and tdTomato^neg^ eGFP^+^ cells also distribute more strongly into G6 than G3, and thus it may be more heterogenous overall. Compared to G6, G3 is enriched for expression of larger and/or distinct sets of genes encoding ECM-remodeling molecules and cell adhesion molecules, in addition to the already described chemokines, interferon response pathway targets, cytokines, and cytokine receptors. Compared to all other groups, G3 is enriched for expression of the largest set of different chemokine genes, which endow it with the unique ability to recruit and organize B and T_FH_ cells through expression of *Cxcl13*, *Ch25h*, and *Cyp7b1*, and to work in conjunction with other groups to recruit Teff and NK cells through expression of *Cxcl10* and *Cxcl16*, to recruit monocytes and neutrophils through expression of *Ccl7*, *Ccl2*, and *Cxcl5*. G3 also is enriched for expression of the largest set of cytokine receptor genes, placing it in a position to respond to both pro- and anti-inflammatory cytokines (Type I IFN, IL1, IL6, OSM, TGFβ, FasL, TWEAK) as well as those associated with Th2 cells (IL4, IL13). G3 is second only to G5 in its enriched expression of genes encoding MHC-II pathway components and interferon response pathway targets. We suggest that this population, which we have not seen identified in other work, serves as an immune orchestrator, and thereby a central organizer, of tumor-associated TLS.Several chemokines have been implicated in human studies as drivers of TLS development, including CXCL9 (36), CXCL10 (98), CCL19 (38–41), CCL20 (99), CCL21 (37), and CXCL13 (31–33, 35–37). Based on our prior work (9, 11, 14, 69, 100, 101), we suggest that these studies actually identify distinct mechanistic elements that underpin TLS development, without necessarily being direct drivers. We have shown that TLS development depends on an initial recruitment of CXCR3^+^ Type I Teff into the tumor through endothelial cell display of CXCL9 and CXCL10. These Teff stimulate the upregulation of PNAd on endothelial cells and CCL19 and CCL21 by CAF, enabling recruitment of CCR7^+^ naïve, and potentially, resting memory, T and B cells. Although we have no direct evidence, it seems likely that CCL20 can serve a similar function for CCR6^+^ T and B cells at either of these two steps. Based on the enriched expression of *Cxcl9* and *Ccl19* shown in the present study, we suggest that this initial recruitment of CXCR3^+^ Teff and CCR7^+^ T and B cells is mediated by CAF in groups G1 and G2, although G3 and G6 may also play a role in Teff recruitment through their enriched expression of *Cxcl10*. In SC tumors, this process does not proceed further, and these tumors contain only a small number of disorganized B cells. By contrast, in IP tumors, CXCL13 mediates the organization and survival of a much larger number of B cells. As we have shown here, the source of this activity is CAF, most likely those in the G3 group. Thus, while cells in G3 mediate the construction of the TLS, the complete sequence of TLS development would involve contributions from CAF in groups G1 and G2 that recruit the TLS building blocks. Applying this understanding to the works above, in which other chemokines are implicated as TLS drivers, we suggest that CXCL9, CXCL10, CCL19, CCL20, and CCL21 each are involved in recruiting one or more TLS building blocks that are essential for TLS development, without involvement in immediate formation of the structure itself.

A remaining question concerns the relevance of the other CAF groups to TLS development. Expression of *April* and *Baff*, which we hypothesize to be important in enhancing B cell survival in TLS containing tumors, was enriched in G0, G5, G1, and G2, respectively, but the extents of enrichment and fractions of expressing cells were small. Expression of genes encoding proteins involved in cell adhesion and junction formation, including VCAM1, which we have associated with CAF in TLS (11), was enriched in G6, G4, and G5, in addition to G3, suggesting that these CAF groups may create scaffolding on which immune cells become organized. These same groups also express *Cxcl10* and *Cxcl16*, and thus have the potential, together with *Cxcl9* expressing G1 and G2 cells, to recruit both Teff and NK cells. CXCL16 has also been shown to recruit monocytes (102) and a subset of Treg cells (103), and each of these chemokines also can affect the differentiation of the cells that they engage with. Perhaps most interesting is the functional consequence of enriched expression of both MHC-II and MHC-I pathway genes by G5, and to a lesser extent, G3. Prior work has suggested that MHC-II expressing CAF can be either immunogenic, tolerogenic, or seemingly neutral (104–108). Neither G5 or G3 expresses costimulatory ligands that would enable effective activation of resting naïve or memory T cells, but G3 shows enriched expression of *Cd274* (PD-L1), *Cd276* (B7-H3), and *Pvr*, a ligand for TIGIT. A similar fraction of G5 cells expresses *Cd274*, but at a somewhat lower level that is not enriched, and instead, G5 is significantly enriched for expression of *CD200*. As MHC-II molecules are ligands for Lag3, they may act as inhibitory molecules as well as antigen presenters. In addition, G5, together with G6 and G3, expressed several MHC class Ib molecules. The H2-Q6 and -Q7 gene products are non-classical MHC-Ib (Qa-2) molecules analogous to human HLA-G, which is involved in protection of the fetus by inhibiting maternal NK-cell mediated lysis (109). H2-T22 and -T23 are non-classical MHC-Ib (Qa-1) molecules analogous to human HLA-E, and engage with both inhibitory and activating NKG2 receptors on NK cells and present antigens to a subset of T cells, including γδ T cells (109–111). Finally, H2-M3 presents N-formylated peptides of bacterial and mitochondrial origin to T cells (112, 113). While the ability of CAF to acquire and present antigens in tumors remains largely unknown (but see ref (104). these results suggest that G5, G3, and G6 may act to constrain activation of effector T cells and/or promote activation of Treg or induce anergy. Demonstration of this, and the consequences in the context of TLS, remain to be explored.

## 4 Materials and methods

### 4.1 Animal models

C57BL/6 mice were from Charles River/NCI. R26R-eGFP^flox^ (B6;129-*Gt(ROSA)26Sor^tm2Sho^*/J) and R26R-iDTR^flox^ (C57BL/6-*Gt(ROSA)26Sor^tm1(HBEGF)Awai^*/J) mice were obtained from Jackson Laboratories. Cxcl13-Cre/tdTomato mice have been described (44). All mice were bred and maintained in specific pathogen-free conditions at the University of Virginia. Both male and female mice were used in experiments. All protocols and experiments were approved by the University of Virginia Institutional Animal Care and Use Committee.

### 4.2 Tumor models

B16-F1 mouse melanoma cells were obtained from American Type Culture Collection (ATCC). The generation of B16-OVA has been described (114). B16-OVA tumor cells were cultured at 37°C and 5% carbon dioxide in RPMI-1640 (Corning) supplemented to a final concentration of 10% (v/v) fetal bovine serum (FBS) (Sigma), 2 mM L-glutamine (ThermoFisher Scientific) and 15 mM HEPES (ThermoFisher Scientific). Tumor cells (4 x 10^5^) in 200 μL phosphate-buffered saline were IP or SC (loose neck scruff) injected into recipient mice and allowed to establish for 14 days prior to harvest. In some experiments, diphtheria toxin (Sigma) (400ng) was injected IP into Cxcl13-cre-tdTomato and Cxcl13-cre-tdTomato X R26R-iDTR^flox^ mice beginning 7 days after tumor implantation, and then daily until tumor harvest. At endpoint, animals were first anesthetized with Ketamine (50–70 mg/kg)/Dexmedetomidine (0.25-0.5 mg/kg) injected IP and then euthanized by cervical dislocation under anesthesia.

### 4.3 Tumor digestion and cell enrichment for analytical flow cytometry

All tumors were weighted after excision, and tumor weight was used to normalize cell count data among tumors. Resected tumors were harvested in DMEM supplemented with 2% FBS, 0.4 mg/ml collagenase dispase, 0.1 mg/ml collagenase P and 50 μg/mL DNase (all from Sigma), 15 mM HEPES, 2 mM L-glutamine, 10 mM sodium pyruvate, 1X essential and non-essential amino acids, gentamicin (1 μg/mL) (all from Gibco). Tumors were minced and digested with a solution containing 0.1 mg/ml DNase I (Sigma), 0.8 mg/ml Collagenase Dispase (Sigma), and 0.2 mg/ml Collagenase P (Sigma) for 15 minutes at 37°C. Every 5 minutes, suspensions were pipetted up-and-down several times. Thereafter, suspensions were filtered through 70 μm mesh (Miltenyi) and depleted of red blood cells by Red Blood Cell Lysing Buffer Hybri-Max (Sigma) according to manufacturer’s instructions. Hematopoietic cells were separated using CD45 MicroBeads mouse (Miltenyi Biotec) on an AutoMACS Pro Separator (Miltenyi Biotec) according to the manufacturer’s instructions. CAF were enriched from CD45-depleted suspensions using biotinylated anti-PDPN (Biolegend) and anti-biotin magnetic beads (Miltenyi Biotec) on an AutoMACS Pro Separator (Miltenyi Biotec) according to the manufacturer’s instructions. Cells were Fc blocked with anti-CD16/CD32 (BioXcell) and stained with Live/Dead Fixable Aqua (ThermoFisher Scientific) in PBS for 20 min at 4 °C.

### 4.4 Tumor digestion and cell enrichment for scRNA seq analysis

Resected tumors were collected in RPMI-1640 (Corning) supplemented with 2% FBS (Sigma), 15 mM HEPES, 2 mM L-glutamine, 1 mM sodium pyruvate, 1X essential and non-essential amino acids, gentamicin (all from Gibco), transferred into RPMI-1640 containing 0.4 mg/ml collagenase II (Fisher/Worthington), 0.1 mg/ml collagenase-dispase, and 50 μg/mL DNase I, minced into small fragments with scissors, and digested for 40 min at 37°C. Digested cell suspensions were homogenized, filtered through 70 μm mesh (Miltenyi Biotec) and depleted of red blood cells with Red Blood Cell Lysing Buffer Hybri-Max at RT for 10 min. Cells were washed and stained with anti-CD16/CD32 and biotinylated anti-PDPN for 1 hour in Awesome MACS buffer [PBS containing 0.5% BSA (Sigma Aldrich), 20mM EDTA (Fisher), 2.5mM dextrose (Sigma Aldrich), 2 mM L-glutamine, 1 mM sodium pyruvate, and 1X essential and non-essential amino acids]. PDPN^+^ cells were enriched using anti-biotin magnetic beads on LS-MACS manual columns (Miltenyi Biotec), and stained with anti-CD45 (Biolegend), anti-CD31 (eBio), and anti-PDPN (Biolegend) in Awesome MACS buffer for 30 min. After staining cells were resuspended in Awesome MACS containing DAPI (0.2 µg/ml) and sorted as described below.

### 4.5 Analytical flow cytometry and cell sorting

For analytical flow cytometry, cell surface staining was done in Awesome MACS containing 2% FBS, 2 mM EDTA (Sigma), and 2 mM NaN_3_ (Sigma) for 15 minutes at 4°C. Samples were run on a FACSCanto II (BD) or Attune NxT (ThermoFisher/Invitrogen) and analyzed using FlowJo Software (BD Bioscience). In all experiments, CAF were defined as live, singlet, Ter119^neg^ (Biolegend), CD45^neg^ (Biolegend), CD31^neg^ (Biolegend), PDPN^hi^ (Biolegend) cells. B16 tumor cells express ten-fold lower levels of PDPN. Thus, only PDPN^hi^ cells were analyzed as CAF. Immune cell populations were defined as CD19^+^ (Biolegend) B cells, CD4^+^ (ThermoFisher) or CD8^+^ (ThermoFisher) T cells, and F4/80^+^ (ThermoFisher) macrophages. Gating controls for these markers were FMO. In experiments involving eGFP, the gating control for tdTomato was a mixture of cells from eGFP mice and B16-M1-GFP expressing cells. In experiments involving iDTR, R26R-iDTR^flox^ mice were used as the gating control for tdTomato.

For scRNA-seq experiments, cells were sorted using a BD Influx System (Becton Dickinson) into BSA-pretreated Eppendorf tubes containing 4% ultra-pure BSA (Sigma) in PBS. CD45^neg^ CD31^neg^ PDPN^hi^ CAF were sorted into tdTomato^neg^ eGFP^neg^, tdTomato^+^ eGFP^ne^g, tdTomato^+^ eGFP^+^, and tdTomato^neg^ eGFP^+^ subpopulations. Viable cells were enumerated using a Cell Drop instrument (Denovix). Live sorted subpopulations were processed for RNA extraction and indexed libraries prepared using the NEBNext Ultra II Directional RNA Library Prep Kit. Single cells were processed using the 10X Genomics Chromium Next GEM Single Cell 3' Reagent Kits v3.1 (Dual Index) to the vendor’ specifications. Illumina NextSeq 2000 were used to sequence the bulk and single cel libraires according to validated standard operating procedures established by the Genome Analysis and Technology Core, RRID: SCR_018883.

### 4.6 Statistical analysis

Statistical details of each experiment in this work are reported in the main and supplementary figure legends. Normality of data distribution was determined by D’Agostino-Pearson omnibus normality test and variance between groups was assessed by the *F*-test. P-values for the comparison between two or more independent groups were calculated by Welch’s t-test and Kruskal-Wallis *h*-test with Dunn’s post-test, respectively. In most cases, error bars shown in graphical data represents mean ± standard deviation for normally distributed data or median ± IQR for non-normal data. P<0.05 was considered statistically significant. All graphs and statistics were calculated using either Graph Pad Prism version 7.0 (**Figures 1**, **2**; **Supplementary Figure 1A**), or R version 4.4.0 (all other presented data).

### 4.7 scRNA-seq analysis

Raw gene expression matrices from PDPN^+^ tdTomato/GFP-sorted cells were merged and used to create a Seurat object using the Seurat 5.0 R package (115). The object was filtered to remove cells with < 500 features or > 6000 features, as well as those with >10% mitochondrial DNA. Raw counts were normalized and the 2000 most variable features were identified using the FindVariableFeatures Seurat command. Normalized counts were scaled and a principal component analysis was conducted using the highly variable features to identify the 50 top principal components. A K nearest-neighbor graph was constructed using the top 25 principal components and used to identify cell clusters. A uniform manifold approximation and projection (UMAP) plot for visualization was created using these clusters. Differential gene expression was used to identify cell types within each cluster. Briefly, a Wilcoxon Ranked Sum test was run using Seurat’s FindAllMarkers function. Enriched genes were used to identify fibroblast and non-fibroblast clusters. The non-fibroblast clusters were removed, and remaining fibroblast cells were reclustered using the same procedure described above for further analysis and definition of fibroblast groups.

### 4.8 Fibroblast group characterization

Fibroblast groups were profiled for function using pathway analysis and subsequent differential gene analysis. Group-specific pathway analysis was performed using the Single Cell Pathway Analysis (SCPA) R package (116). Briefly, for each group, 1200 cells were randomly selected and compared for pathway enrichment using SCPA against 1200 cells randomly selected from all other groups. Pathways were taken from the GO msigDB mouse collection (117–119). Highly enriched pathways were then used to identify differentially expressed genes that informed fibroblast group functional differences. Pathways with highly significant positive enrichment were used to identify functions differentiating each cell group from other groups. Representative pathways best defining applicable functions were selected for display in **Figure 3D**.

To further define cell group behaviors, differential gene expression was performed for pathways identified as highly significant to determine individual genes driving pathway enrichment. Individual genes with significant enrichment for one or more cell groups were selected for dot plots. Genes enriched in related pathways were grouped into the same dot plots. Expression values were normalized across groups, with dot size representing the percentage of cells in a given group expressing the gene.

## Data Availability

The original contributions presented in the study are publicly available. This data can be found here: GEO database, accession number GSE338793.

## References

[1] Stranford S Ruddle NH . Follicular dendritic cells, conduits, lymphatic vessels, and high endothelial venules in tertiary lymphoid organs: Parallels with lymph node stroma. Front Immunol. (2012) 3:350. doi: 10.3389/fimmu.2012.00350 23230435 PMC3515885

[2] Jones GW Hill DG Jones SA . Understanding immune cells in tertiary lymphoid organ development: It is all starting to come together. Front Immunol. (2016) 7:401. doi: 10.3389/fimmu.2016.00401 27752256 PMC5046062

[3] Hiraoka N Ino Y Yamazaki-Itoh R . Tertiary lymphoid organs in cancer tissues. Front Immunol. (2016) 7:244. doi: 10.3389/fimmu.2016.00244 27446075 PMC4916185

[4] Colbeck EJ Ager A Gallimore A Jones GW . Tertiary lymphoid structures in cancer: Drivers of antitumor immunity, immunosuppression, or bystander sentinels in disease? Front Immunol. (2017) 8:1830. doi: 10.3389/fimmu.2017.01830 29312327 PMC5742143

[5] Sautès-Fridman C Petitprez F Calderaro J Fridman WH . Tertiary lymphoid structures in the era of cancer immunotherapy. Nat Rev Cancer. (2019) 19:307–25. doi: 10.1038/s41568-019-0144-6 31092904

[6] Rodriguez AB Engelhard VH . Insights into tumor-associated tertiary lymphoid structures: Novel targets for antitumor immunity and cancer immunotherapy. Cancer Immunol Res. (2020) 8:1338–45. doi: 10.1158/2326-6066.CIR-20-0432 33139300 PMC7643396

[7] Engelhard VH Rodriguez AB Mauldin IS Woods AN Peske JD Slingluff CL . Immune cell infiltration and tertiary lymphoid structures as determinants of antitumor immunity. J Immunol. (2018) 200:432–42. doi: 10.4049/jimmunol.1701269 29311385 PMC5777336

[8] Joshi NS Akama-Garren EH Lu Y Lee D-Y Chang GP Li A . Regulatory T cells in tumor-associated tertiary lymphoid structures suppress anti-tumor T cell responses. Immunity. (2015) 43:579–90. doi: 10.1016/j.immuni.2015.08.006 26341400 PMC4826619

[9] Peske JD Thompson ED Gemta L Baylis RA Fu Y-X Engelhard VH . Effector lymphocyte-induced lymph node-like vasculature enables naive T-cell entry into tumours and enhanced anti-tumour immunity. Nat Commun. (2015) 6:7114. doi: 10.1038/ncomms8114 25968334 PMC4435831

[10] Rodriguez AB Peske JD Engelhard VH . Identification and characterization of tertiary lymphoid structures in murine melanoma. Methods Mol Biol. (2018) 1845:241–57. doi: 10.1007/978-1-4939-8709-2_14 30141017 PMC6269110

[11] Rodriguez AB Peske JD Woods AN Leick KM Mauldin IS Meneveau MO . Immune mechanisms orchestrate tertiary lymphoid structures in tumors via cancer-associated fibroblasts. Cell Rep. (2021) 36:109422. doi: 10.1016/j.celrep.2021.109422 34289373 PMC8362934

[12] Overacre-Delgoffe AE Bumgarner HJ Cillo AR Burr AHP Tometich JT Bhattacharjee A . Microbiota-specific T follicular helper cells drive tertiary lymphoid structures and anti-tumor immunity against colorectal cancer. Immunity. (2021) 54:2812–2824.e4. doi: 10.1016/j.immuni.2021.11.003 34861182 PMC8865366

[13] van Hooren L Vaccaro A Ramachandran M Vazaios K Libard S van de Walle T . Agonistic CD40 therapy induces tertiary lymphoid structures but impairs responses to checkpoint blockade in glioma. Nat Commun. (2021) 12:4127. doi: 10.1038/s41467-021-24347-7 34226552 PMC8257767

[14] Thompson ED Enriquez HL Fu Y-X Engelhard VH . Tumor masses support naive T cell infiltration, activation, and differentiation into effectors. J Exp Med. (2010) 207:1791–804. doi: 10.1084/jem.20092454 20660615 PMC2916130

[15] Kraman M Bambrough PJ Arnold JN Roberts EW Magiera L Jones JO . Suppression of antitumor immunity by stromal cells expressing fibroblast activation protein-alpha. Science. (2010) 330:827–30. doi: 10.1126/science.1195300 21051638

[16] Liu T Han C Wang S Fang P Ma Z Xu L . Cancer-associated fibroblasts: An emerging target of anti-cancer immunotherapy. J Hematol Oncol. (2019) 12:86. doi: 10.1186/s13045-019-0770-1 31462327 PMC6714445

[17] Yamauchi M Barker TH Gibbons DL Kurie JM . The fibrotic tumor stroma. J Clin Invest. (2018) 128:16–25. doi: 10.1172/JCI93554 29293090 PMC5749516

[18] Denton AE Roberts EW Fearon DT . Stromal cells in the tumor microenvironment. In: Owens BMJ Lakins MA , editors. Stromal Immunology. Springer International Publishing, Cham (2018). p. 99–114. doi: 10.1007/978-3-319-78127-3_6 30155624

[19] Sahai E Astsaturov I Cukierman E DeNardo DG Egeblad M Evans RM . A framework for advancing our understanding of cancer-associated fibroblasts. Nat Rev Cancer. (2020) 20:174–86. doi: 10.1038/s41568-019-0238-1 31980749 PMC7046529

[20] Chen Y McAndrews KM Kalluri R . Clinical and therapeutic relevance of cancer-associated fibroblasts. Nat Rev Clin Oncol. (2021) 18:792–804. doi: 10.1038/s41571-021-00546-5 34489603 PMC8791784

[21] Caligiuri G Tuveson DA . Activated fibroblasts in cancer: Perspectives and challenges. Cancer Cell. (2023) 41:434–49. doi: 10.1016/j.ccell.2023.02.015 36917949 PMC11022589

[22] Chhabra Y Weeraratna AT . Fibroblasts in cancer: Unity in heterogeneity. Cell. (2023) 186:1580–609. doi: 10.1016/j.cell.2023.03.016 37059066 PMC11422789

[23] Lavie D Ben-Shmuel A Erez N Scherz-Shouval R . Cancer-associated fibroblasts in the single-cell era. Nat Cancer. (2022) 3:793–807. doi: 10.1038/s43018-022-00411-z 35883004 PMC7613625

[24] Chen B Chan WN Xie F Mui CW Liu X Cheung AHK . The molecular classification of cancer‐associated fibroblasts on a pan‐cancer single‐cell transcriptional atlas. Clin Transl Med. (2023) 13:e1516. doi: 10.1002/ctm2.1516 38148640 PMC10751516

[25] Kazakova AN Lukina MM Anufrieva KS Bekbaeva IV Ivanova OM Shnaider PV . Exploring the diversity of cancer-associated fibroblasts: Insights into mechanisms of drug resistance. Front Cell Dev Biol. (2024) 12:1403122. doi: 10.3389/fcell.2024.1403122 38818409 PMC11137237

[26] Flynn JM Thadani N Gallagher EE Azzaro I Bodnar CM McCarty CP . Plasticity and functional heterogeneity of cancer-associated fibroblasts. Cancer Res. (2025) 85:3378–98. doi: 10.1158/0008-5472.CAN-24-3037 40729489 PMC12371731

[27] Paraskeva C Stavropoulou A Koliaraki V . Cancer-associated fibroblasts: Recent advances and therapeutic implications. Curr Opin Cell Biol. (2026) 98:102601. doi: 10.1016/j.ceb.2025.102601 41330025

[28] Thommen DS Koelzer VH Herzig P Roller A Trefny M Dimeloe S . A transcriptionally and functionally distinct PD-1+ CD8+ T cell pool with predictive potential in non-small-cell lung cancer treated with PD-1 blockade. Nat Med. (2018) 24:994–1004. doi: 10.1038/s41591-018-0057-z 29892065 PMC6110381

[29] Li H van der Leun AM Yofe I Lubling Y Gelbard-Solodkin D van Akkooi ACJ . Dysfunctional CD8 T cells form a proliferative, dynamically regulated compartment within human melanoma. Cell. (2019) 176:775–789.e18. doi: 10.1016/j.cell.2018.11.043 30595452 PMC7253294

[30] Workel HH Lubbers JM Arnold R Prins TM Van Der Vlies P De Lange K . A transcriptionally distinct CXCL13+CD103+CD8+ T-cell population is associated with B-cell recruitment and neoantigen load in human cancer. Cancer Immunol Res. (2019) 7:784–96. doi: 10.1158/2326-6066.CIR-18-0517 30872264

[31] Ukita M Hamanishi J Yoshitomi H Yamanoi K Takamatsu S Ueda A . CXCL13-producing CD4+ T cells accumulate in the early phase of tertiary lymphoid structures in ovarian cancer. JCI Insight. (2022) 7:e157215. doi: 10.1172/jci.insight.157215 35552285 PMC9309049

[32] Goubet A-G Lordello L Alves Costa Silva C Peguillet I Gazzano M Mbogning-Fonkou MD . Escherichia coli-specific CXCL13-producing TFH are associated with clinical efficacy of neoadjuvant PD-1 blockade against muscle-invasive bladder cancer. Cancer Discov. (2022) 12:2280–307. doi: 10.1158/2159-8290.CD-22-0201 35929803

[33] Chaurio RA Anadon CM Lee Costich T Payne KK Biswas S Harro CM . TGF-β-mediated silencing of genomic organizer SATB1 promotes Tfh cell differentiation and formation of intra-tumoral tertiary lymphoid structures. Immunity. (2022) 55:115–128.e9. doi: 10.1016/j.immuni.2021.12.007 35021053 PMC8852221

[34] Sorin M Karimi E Rezanejad M Yu MW Desharnais L McDowell SAC . Single-cell spatial landscape of immunotherapy response reveals mechanisms of CXCL13 enhanced antitumor immunity. J Immunother Cancer. (2023) 11:e005545. doi: 10.1136/jitc-2022-005545 36725085 PMC9896310

[35] Chen Y Wu Y Zhao Z Wen L Wu M Song D . Retrospective study on the correlation between CXCL13, immune infiltration, and tertiary lymphoid structures in cutaneous squamous cell carcinoma. PeerJ. (2025) 13:e19398. doi: 10.7717/peerj.19398 40352278 PMC12065455

[36] Nagase Y Kodama M Aimono E Nakamura K Takamatsu R Abe K . CXCL9 and CXCL13 shape endometrial cancer immune-activated microenvironment via tertiary lymphoid structure formation. Cancer Sci. (2025) 116:1193–202. doi: 10.1111/cas.16371 39960836 PMC12044659

[37] Yoshimitsu M Nakamura M Kano S Magara T Kato H Sakai A . CXCL13 and CCL21 induce tertiary lymphoid structures and enhance the efficacy of immunotherapy for melanoma. Cancer Sci. (2025) 116:2075–85. doi: 10.1111/cas.70105 40393449 PMC12317389

[38] Zhang Y Liu G Zeng Q Wu W Lei K Zhang C . CCL19-producing fibroblasts promote tertiary lymphoid structure formation enhancing anti-tumor IgG response in colorectal cancer liver metastasis. Cancer Cell. (2024) 42:1370–1385.e9. doi: 10.1016/j.ccell.2024.07.006 39137726

[39] Jenkins BH Tracy I Rodrigues MFSD Smith MJL Martinez BR Edmond M . Single cell and spatial analysis of immune-hot and immune-cold tumours identifies fibroblast subtypes associated with distinct immunological niches and positive immunotherapy response. Mol Cancer. (2025) 24:3. doi: 10.1186/s12943-024-02191-9 39757146 PMC11702232

[40] Xu W Weng J Zhao Y Xie P Xu M Liu S . FMO2(+) cancer-associated fibroblasts sensitize anti-PD-1 therapy in patients with hepatocellular carcinoma. J Immunother Cancer. (2025) 13:e011648. doi: 10.1136/jitc-2025-011648 40316306 PMC12049961

[41] Tang Q-Y Zhang Y-J Wu Z-Y Zhang T-Y Song X-L Zhu Y-D . Single-cell profiling unveils the immuno-favorable tumor microenvironment remodeling after successful neoadjuvant therapy for advanced gallbladder cancer. Int J Surg Lond Engl. (2025) 112:4110–4124. doi: 10.1097/JS9.0000000000003883 41202319

[42] Ansel KM Harris RBS Cyster JG . CXCL13 is required for B1 cell homing, natural antibody production, and body cavity immunity. Immunity. (2002) 16:67–76. doi: 10.1016/S1074-7613(01)00257-6 11825566

[43] Mackay F Schneider P Rennert P Browning J . BAFF and APRIL: A tutorial on B cell survival. Annu Rev Immunol. (2003) 21:231–64. doi: 10.1146/annurev.immunol.21.120601.141152 12427767

[44] Onder L Mörbe U Pikor N Novkovic M Cheng H-W Hehlgans T . Lymphatic endothelial cells control initiation of lymph node organogenesis. Immunity. (2017) 47:80–92.e4. doi: 10.1016/j.immuni.2017.05.008 28709801

[45] Buechler MB Pradhan RN Krishnamurty AT Cox C Calviello AK Wang AW . Cross-tissue organization of the fibroblast lineage. Nature. (2021) 593:575–9. doi: 10.1038/s41586-021-03549-5 33981032

[46] Croizer H Mhaidly R Kieffer Y Gentric G Djerroudi L Leclere R . Deciphering the spatial landscape and plasticity of immunosuppressive fibroblasts in breast cancer. Nat Commun. (2024) 15:2806. doi: 10.1038/s41467-024-47068-z 38561380 PMC10984943

[47] Xiao Z Todd L Huang L Noguera-Ortega E Lu Z Huang L . Desmoplastic stroma restricts T cell extravasation and mediates immune exclusion and immunosuppression in solid tumors. Nat Commun. (2023) 14:5110. doi: 10.1038/s41467-023-40850-5 37607999 PMC10444764

[48] De Oliveira Macena Y Cezar MEN Lira CBF De Oliveira LBDM Almeida TN Costa ADAV . The roles of periostin derived from cancer-associated fibroblasts in tumor progression and treatment response. Cancer Metastasis Rev. (2025) 44:11. doi: 10.1007/s10555-024-10233-3 39614015

[49] Lei J Liu Y Yuan S Yuan X Yuan Q . Periostin-integrin signaling in hepatocellular carcinoma: From biological function to clinical application. Front Cell Dev Biol. (2025) 13:1520739. doi: 10.3389/fcell.2025.1520739 41018265 PMC12463895

[50] Danen E . Integrins: an overview of structural and functional aspects. In: Madame curie bioscience database. Landes Bioscience, Austin (TX (2000).

[51] Humphries JD Byron A Humphries MJ . Integrin ligands at a glance. J Cell Sci. (2006) 119:3901–3. doi: 10.1242/jcs.03098 16988024 PMC3380273

[52] Petruzzelli L Takami M Humes HD . Structure and function of cell adhesion molecules. Am J Med. (1999) 106:467–76. doi: 10.1016/S0002-9343(99)00058-3 10225251

[53] Kobayashi H Boelte K Lin PC . Endothelial cell adhesion molecules and cancer progression. Curr Med Chem. (2007) 14:377–86. doi: 10.2174/092986707779941032 17305540

[54] DeGrendele HC Estess P Siegelman MH . Requirement for CD44 in activated T cell extravasation into an inflammatory site. Science. (1997) 278:672–5. doi: 10.1126/science.278.5338.672 9381175

[55] Meran S Martin J Luo DD Steadman R Phillips A . Interleukin-1β induces hyaluronan and CD44-dependent cell protrusions that facilitate fibroblast-monocyte binding. Am J Pathol. (2013) 182:2223–40. doi: 10.1016/j.ajpath.2013.02.038 23583650

[56] Barreiro O Yáñez-Mó M Serrador JM Montoya MC Vicente-Manzanares M Tejedor R . Dynamic interaction of VCAM-1 and ICAM-1 with moesin and ezrin in a novel endothelial docking structure for adherent leukocytes. J Cell Biol. (2002) 157:1233–45. doi: 10.1083/jcb.200112126 12082081 PMC2173557

[57] Thorne RF Legg JW Isacke CM . The role of the CD44 transmembrane and cytoplasmic domains in co-ordinating adhesive and signalling events. J Cell Sci. (2004) 117:373–80. doi: 10.1242/jcs.00954 14702383

[58] Darling TK Lamb TJ . Emerging roles for Eph receptors and ephrin ligands in immunity. Front Immunol. (2019) 10:1473. doi: 10.3389/fimmu.2019.01473 31333644 PMC6620610

[59] Shapiro L Fannon AM Kwong PD Thompson A Lehmann MS Grübel G . Structural basis of cell-cell adhesion by cadherins. Nature. (1995) 374:327–37. doi: 10.1038/374327a0 7885471

[60] Balabanian K Lagane B Infantino S Chow KYC Harriague J Moepps B . The chemokine SDF-1/CXCL12 binds to and signals through the orphan receptor RDC1 in T lymphocytes. J Biol Chem. (2005) 280:35760–6. doi: 10.1074/jbc.M508234200 16107333

[61] Burns JM Summers BC Wang Y Melikian A Berahovich R Miao Z . A novel chemokine receptor for SDF-1 and I-TAC involved in cell survival, cell adhesion, and tumor development. J Exp Med. (2006) 203:2201–13. doi: 10.1084/jem.20052144 16940167 PMC2118398

[62] Rajagopal S Kim J Ahn S Craig S Lam CM Gerard NP . Beta-arrestin- but not G protein-mediated signaling by the “decoy” receptor CXCR7. Proc Natl Acad Sci USA. (2010) 107:628–32. doi: 10.1073/pnas.0912852107 20018651 PMC2818968

[63] Sun X Cheng G Hao M Zheng J Zhou X Zhang J . CXCL12 / CXCR4 / CXCR7 chemokine axis and cancer progression. Cancer Metastasis Rev. (2010) 29:709–22. doi: 10.1007/s10555-010-9256-x 20839032 PMC3175097

[64] Salcedo R Young HA Ponce ML Ward JM Kleinman HK Murphy WJ . Eotaxin (CCL11) induces *in vivo* angiogenic responses by human CCR3+ endothelial cells1. J Immunol. (2001) 166:7571–8. doi: 10.4049/jimmunol.166.12.7571 11390513

[65] Pawig L Klasen C Weber C Bernhagen J Noels H . Diversity and inter-connections in the CXCR4 chemokine receptor/ligand family: molecular perspectives. Front Immunol. (2015) 6. doi: 10.3389/fimmu.2015.00429 26347749 PMC4543903

[66] Janssens R Struyf S Proost P . Pathological roles of the homeostatic chemokine CXCL12. Cytokine Growth Fact Rev. (2018) 44:51–68. doi: 10.1016/j.cytogfr.2018.10.004 30396776

[67] Bianchi ME Mezzapelle R . The chemokine receptor CXCR4 in cell proliferation and tissue regeneration. Front Immunol. (2020) 11:2109. doi: 10.3389/fimmu.2020.02109 32983169 PMC7484992

[68] Giorgiutti S Rottura J Korganow A-S Gies V . CXCR4: from B-cell development to B cell–mediated diseases. Life Sci Alliance. (2024) 7:e202302465. doi: 10.26508/lsa.202302465 38519141 PMC10961644

[69] Woods AN Wilson AL Srivinisan N Zeng J Dutta AB Peske JD . Differential expression of homing receptor ligands on tumor-associated vasculature that control CD8 effector T-cell entry. Cancer Immunol Res. (2017) 5:1062–73. doi: 10.1158/2326-6066.CIR-17-0190 29097419 PMC6069521

[70] Yi T Wang X Kelly LM An J Xu Y Sailer AW . Oxysterol gradient generation by lymphoid stromal cells guides activated B cell movement during humoral responses. Immunity. (2012) 37:535–48. doi: 10.1016/j.immuni.2012.06.015 22999953 PMC3465460

[71] Ozga AJ Chow MT Lopes ME Servis RL Pilato MD Dehio P . CXCL10 chemokine regulates heterogeneity of the CD8+ T cell response and viral set point during chronic infection. Immunity. (2022) 55:82–97.e8. doi: 10.1016/j.immuni.2021.11.002 34847356 PMC8755631

[72] Matloubian M David A Engel S Ryan JE Cyster JG . A transmembrane CXC chemokine is a ligand for HIV-coreceptor Bonzo. Nat Immunol. (2000) 1:298–304. doi: 10.1038/79738 11017100

[73] Hara T Katakai T Lee J-H Nambu Y Nakajima-Nagata N Gonda H . A transmembrane chemokine, CXC chemokine ligand 16, expressed by lymph node fibroblastic reticular cells has the potential to regulate T cell migration and adhesion. Int Immunol. (2006) 18:301–11. doi: 10.1093/intimm/dxh369 16410312

[74] Shin DS Ribas A . The evolution of checkpoint blockade as a cancer therapy: what’s here, what’s next? Curr Opin Immunol. (2015) 33:23–35. doi: 10.1016/j.coi.2015.01.006 25621841

[75] Schnell A Bod L Madi A Kuchroo VK . The yin and yang of co-inhibitory receptors: toward anti-tumor immunity without autoimmunity. Cell Res. (2020) 30:285–99. doi: 10.1038/s41422-020-0277-x 31974523 PMC7118128

[76] Zaitseva O Hoffmann A Otto C Wajant H . Targeting fibroblast growth factor (FGF)-inducible 14 (Fn14) for tumor therapy. Front Pharmacol. (2022) 13:935086. doi: 10.3389/fphar.2022.935086 36339601 PMC9634131

[77] Zheng Y Liu W-H Yang B Milman Krentsis I . Primer on fibroblast growth factor 7 (FGF 7). Differentiation. (2024) 139:100801. doi: 10.1016/j.diff.2024.100801 39048474

[78] Achen MG Jeltsch M Kukk E Mäkinen T Vitali A Wilks AF . Vascular endothelial growth factor D (VEGF-D) is a ligand for the tyrosine kinases VEGF receptor 2 (Flk1) and VEGF receptor 3 (Flt4). Proc Natl Acad Sci. (1998) 95:548–53. doi: 10.1073/pnas.95.2.548 9435229 PMC18457

[79] Stanley ER Berg KL Einstein DB Lee PSW Pixley FJ Wang Y . Biology and action of colony-stimulating factor-1. Mol Reprod Dev. (1997) 46:4–10. doi: 10.1002/(SICI)1098-2795(199701)46:1<4::AID-MRD2>3.0.CO;2-V 8981357

[80] Wahl SM . Transforming growth factor-β: innately bipolar. Curr Opin Immunol. (2007) 19:55–62. doi: 10.1016/j.coi.2006.11.008 17137775

[81] Ray S Ju X Sun H Finnerty CC Herndon DN Brasier AR . The IL-6 trans-signaling-STAT3 pathway mediates ECM and cellular proliferation in fibroblasts from hypertrophic scar. J Invest Dermatol. (2013) 133:1212–20. doi: 10.1038/jid.2012.499 23303450 PMC3626764

[82] O’Reilly S Ciechomska M Cant R van Laar JM . Interleukin-6 (IL-6) trans signaling drives a STAT3-dependent pathway that leads to hyperactive transforming growth factor-β (TGF-β) signaling promoting SMAD3 activation and fibrosis via Gremlin protein. J Biol Chem. (2014) 289:9952–60. doi: 10.1074/jbc.M113.545822 24550394 PMC3975039

[83] Yang L Guo P Wang P Wang W Liu J . IL-6/ERK signaling pathway participates in type I IFN-programmed, unconventional M2-like macrophage polarization. Sci Rep. (2023) 13:1827. doi: 10.1038/s41598-022-23721-9 36726024 PMC9892596

[84] Gao X Wang X Yang Q Zhao X Wen W Li G . Tumoral expression of IL-33 inhibits tumor growth and modifies the tumor microenvironment through CD8+ T and NK cells. J Immunol. (2014) 194:438–45. doi: 10.4049/jimmunol.1401344 25429071 PMC4272901

[85] Dominguez D Ye C Geng Z Chen S Fan J Qin L . Exogenous IL-33 restores dendritic cell activation and maturation in established cancer. J Immunol. (2017) 198:1365–75. doi: 10.4049/jimmunol.1501399 28011934 PMC5263113

[86] Shani O Vorobyov T Monteran L Lavie D Cohen N Raz Y . Fibroblast-derived IL33 facilitates breast cancer metastasis by modifying the immune microenvironment and driving type 2 immunity. Cancer Res. (2020) 80:5317–29. doi: 10.1158/0008-5472.CAN-20-2116 33023944 PMC7611300

[87] Sun R Zhao H Gao DS Ni A Li H Chen L . Amphiregulin couples IL1RL1^+^ regulatory T cells and cancer-associated fibroblasts to impede antitumor immunity. Sci Adv. (2023) 9:eadd7399. doi: 10.1126/sciadv.add7399 37611111 PMC10446484

[88] Dai S Zeng H Liu Z Jin K Jiang W Wang Z . Intratumoral CXCL13^+^ CD8^+^ T cell infiltration determines poor clinical outcomes and immunoevasive contexture in patients with clear cell renal cell carcinoma. J Immunother Cancer. (2021) 9:e001823. doi: 10.1136/jitc-2020-001823 33589528 PMC7887366

[89] Ren J Lan T Liu T Liu Y Shao B Men K . CXCL13 as a novel immune checkpoint for regulatory B cells and its role in tumor metastasis. J Immunol. (2022) 208:2425–35. doi: 10.4049/jimmunol.2100341 35437281 PMC9125199

[90] Galbo PM Zang X Zheng D . Molecular features of cancer-associated fibroblast subtypes and their implication on cancer pathogenesis, prognosis, and immunotherapy resistance. Clin Cancer Res. (2021) 27:2636–47. doi: 10.1158/1078-0432.CCR-20-4226 33622705 PMC8102353

[91] Carretta M Thorseth M-L SChina A Agardy DA Johansen AZ Baker KJ . Dissecting tumor microenvironment heterogeneity in syngeneic mouse models: insights on cancer-associated fibroblast phenotypes shaped by infiltrating T cells. Front Immunol. (2024) 14:1320614. doi: 10.3389/fimmu.2023.1320614 38259467 PMC10800379

[92] Chen X Zhou Z Xie L Qiao K Jia Y Liu S . Single-cell resolution spatial analysis of antigen-presenting cancer-associated fibroblast niches. Cancer Cell. (2025) 43:2224–2240.e9. doi: 10.1016/j.ccell.2025.09.001 41005303 PMC12479095

[93] Liu Y Sinjab A Min J Han G Paradiso F Zhang Y . Conserved spatial subtypes and cellular neighborhoods of cancer-associated fibroblasts revealed by single-cell spatial multi-omics. Cancer Cell. (2025) 43:905–924.e6. doi: 10.1016/j.ccell.2025.03.004 40154487 PMC12074878

[94] Cords L Tietscher S Anzeneder T Langwieder C Rees M De Souza N . Cancer-associated fibroblast classification in single-cell and spatial proteomics data. Nat Commun. (2023) 14:4294. doi: 10.1038/s41467-023-39762-1 37463917 PMC10354071

[95] Cumming J Maneshi P Dongre M Alsaed T Dehghan-Nayeri MJ Ling A . Dissecting FAP+ cell diversity in pancreatic cancer uncovers an interferon-response subtype of cancer-associated fibroblasts with tumor-restraining properties. Cancer Res. (2025) 85:2388–411. doi: 10.1158/0008-5472.CAN-23-3252 40215177 PMC12214878

[96] Davidson S Efremova M Riedel A Mahata B Pramanik J Huuhtanen J . Single-cell RNA sequencing reveals a dynamic stromal niche that supports tumor growth. Cell Rep. (2020) 31:107628. doi: 10.1016/j.celrep.2020.107628 32433953 PMC7242909

[97] Forsthuber A Aschenbrenner B Korosec A Jacob T Annusver K Krajic N . Cancer-associated fibroblast subtypes modulate the tumor-immune microenvironment and are associated with skin cancer Malignancy. Nat Commun. (2024) 15:9678. doi: 10.1038/s41467-024-53908-9 39516494 PMC11549091

[98] Zhang M-J Lin W-P Wang Q Wang S Song A Wang Y-Y . Oncolytic herpes simplex virus propagates tertiary lymphoid structure formation via CXCL10/CXCR3 to boost antitumor immunity. Cell Prolif. (2025) 58:e13740. doi: 10.1111/cpr.13740 39219056 PMC11693575

[99] Zhang S Liu H Li X Jiang Y Tang L Liu T . CCL20 secreted by KRT15(high) tumor cells promotes tertiary lymphoid structure formation and enhances anti-PD-1 therapy response in HPV(+)HNSCC. Cell Death Dis. (2025) 17:150. doi: 10.1038/s41419-025-08359-5 41462011 PMC12858956

[100] Peske JD Woods AB Engelhard VH . Control of CD8 T-cell infiltration into tumors by vasculature and microenvironment. Adv Cancer Res. (2015) 128:263–307. doi: 10.1016/bs.acr.2015.05.001 26216636 PMC4638417

[101] Rodriguez AB Parriott G Engelhard VH . Tumor necrosis factor receptor regulation of peripheral node addressin biosynthetic components in tumor endothelial cells. Front Immunol. (2022) 13:1009306. doi: 10.3389/fimmu.2022.1009306 36189308 PMC9520236

[102] Allaoui R Bergenfelz C Mohlin S Hagerling C Salari K Werb Z . Cancer-associated fibroblast-secreted CXCL16 attracts monocytes to promote stroma activation in triple-negative breast cancers. Nat Commun. (2016) 7:13050. doi: 10.1038/ncomms13050 27725631 PMC5062608

[103] Ye L Xiang X Wang Z Zhang S Xue Q Wei X . H3K18 lactylation in cancer-associated fibroblasts drives Malignant pleural effusion progression via TNFR2+ Treg recruitment. Exp Mol Med. (2025) 57:2671–85. doi: 10.1038/s12276-025-01557-3 41310104 PMC12686498

[104] Lakins MA Ghorani E Munir H Martins CP Shields JD . Cancer-associated fibroblasts induce antigen-specific deletion of CD8 + T Cells to protect tumour cells. Nat Commun. (2018) 9:948. doi: 10.1038/s41467-018-03347-0 29507342 PMC5838096

[105] Elyada E Bolisetty M Laise P Flynn WF Courtois ET Burkhart RA . Cross-species single-cell analysis of pancreatic ductal adenocarcinoma reveals antigen-presenting cancer-associated fibroblasts. Cancer Discov. (2019) 9:1102–23. doi: 10.1158/2159-8290.CD-19-0094 31197017 PMC6727976

[106] Friedman G Levi-Galibov O David E Bornstein C Giladi A Dadiani M . Cancer-associated fibroblast compositions change with breast cancer progression linking the ratio of S100A4+ and PDPN+ CAFs to clinical outcome. Nat Cancer. (2020) 1:692–708. doi: 10.1038/s43018-020-0082-y 35122040 PMC7617059

[107] Huang H Wang Z Zhang Y Pradhan RN Ganguly D Chandra R . Mesothelial cell-derived antigen-presenting cancer-associated fibroblasts induce expansion of regulatory T cells in pancreatic cancer. Cancer Cell. (2022) 40:656–673.e7. doi: 10.1016/j.ccell.2022.04.011 35523176 PMC9197998

[108] Kerdidani D Aerakis E Verrou K-M Angelidis I Douka K Maniou M-A . Lung tumor MHCII immunity depends on in situ antigen presentation by fibroblasts. J Exp Med. (2022) 219:e20210815. doi: 10.1084/jem.20210815 35029648 PMC8764966

[109] Sullivan LC Hoare HL McCluskey J Rossjohn J Brooks AG . A structural perspective on MHC class Ib molecules in adaptive immunity. Trends Immunol. (2006) 27:413–20. doi: 10.1016/j.it.2006.07.006 16860610

[110] Fahl SP Coffey F Kain L Zarin P Dunbrack RL Teyton L . Role of a selecting ligand in shaping the murine γδ-TCR repertoire. Proc Natl Acad Sci. (2018) 115:1889–94. doi: 10.1073/pnas.1718328115 29432160 PMC5828614

[111] Früh K Borrow P Gillespie GM McMichael AJ Picker LJ . Targeting MHC-E as a new strategy for vaccines and immunotherapeutics. Nat Rev Immunol. (2026) 26:52–66. doi: 10.1038/s41577-025-01218-6 40903525 PMC13078113

[112] Pamer EG Wang CR Flaherty L Fischer Lindahl K Bevan MJ . H-2M3 presents a listeria monocytogenes peptide to cytotoxic T lymphocytes. Cell. (1992) 70:215–23. doi: 10.1016/0092-8674(92)90097-v 1353418

[113] Stroynowski I Lindahl KF . Antigen presentation by non-classical class I molecules. Curr Opin Immunol. (1994) 6:38–44. doi: 10.1016/0952-7915(94)90031-0 8172679

[114] Hargadon KM Brinkman CC Sheasley-O’Neill SL Nichols LA Bullock TNJ Engelhard VH . Incomplete differentiation of antigen-specific CD8 T Cells in tumor-draining lymph nodes. J Immunol. (2006) 177:6081–90. doi: 10.4049/jimmunol.177.9.6081 17056534

[115] Hao Y Stuart T Kowalski MH Choudhary S Hoffman P Hartman A . Dictionary learning for integrative, multimodal and scalable single-cell analysis. Nat Biotechnol. (2024) 42:293–304. doi: 10.1038/s41587-023-01767-y 37231261 PMC10928517

[116] Bibby JA Agarwal D Freiwald T Kunz N Merle NS West EE . Systematic single-cell pathway analysis to characterize early T cell activation. Cell Rep. (2022) 41:111697. doi: 10.1016/j.celrep.2022.111697 36417885 PMC10704209

[117] Liberzon A Subramanian A Pinchback R Thorvaldsdóttir H Tamayo P Mesirov JP . Molecular signatures database (MSigDB) 3.0. Bioinformatics. (2011) 27:1739–40. doi: 10.1093/bioinformatics/btr260 21546393 PMC3106198

[118] Castanza AS Recla JM Eby D Thorvaldsdóttir H Bult CJ Mesirov JP . Extending support for mouse data in the Molecular Signatures Database (MSigDB). Nat Methods. (2023) 20:1619–20. doi: 10.1038/s41592-023-02014-7 37704782 PMC11397807

[119] Subramanian A Tamayo P Mootha VK Mukherjee S Ebert BL Gillette MA . Gene set enrichment analysis: A knowledge-based approach for interpreting genome-wide expression profiles. Proc Natl Acad Sci. (2005) 102:15545–50. doi: 10.1073/pnas.0506580102 16199517 PMC1239896

