# Supplementary material for: Characterization of cancer-associated fibroblast populations that promote tertiary lymphoid structure formation in murine melanoma tumors
Source: Front Immunol. 2026 Jul 17;17:1857037. doi: 10.3389/fimmu.2026.1857037 (PMC13425456; doi:10.3389/fimmu.2026.1857037)
Supplement: Supplementary file 1 [file Supplementaryfile1.zip › Supplementary Figures and Supplementary Tables 1 and 5.PDF]

Supplementary Table 1: Differential expression of TLS-associated genes among CAF groups

Gene	Group	Avg_log2FC	Pct.1	Pct.2	p_val_adj
Fap	G0	2.08	0.13	0.13	1.00E+00
	**G1**	**1.03**	**0.25**	**0.10**	**1.70E-120**
	**G2**	**0.54**	**0.27**	**0.12**	**1.15E-48**
	G3	-0.82	0.12	0.13	1.00E+00
	G6	-1.66	0.09	0.14	1.46E-16
	G4	-0.31	0.10	0.13	1.00E+00
	G5	-3.38	0.02	0.15	1.47E-86
Vcam1	G0	-2.46	0.10	0.76	0.00E+00
	G1	-0.86	0.62	0.71	5.66E-90
	G2	-0.76	0.75	0.69	5.15E-15
	**G3**	**0.37**	**0.84**	**0.65**	**5.70E-93**
	**G6**	**0.30**	**0.87**	**0.65**	**3.38E-99**
	**G4**	**0.61**	**0.71**	**0.69**	**3.74E-34**
	**G5**	**0.40**	**0.69**	**0.69**	**3.93E-15**
Tnfsf13 (April)	**G0**	**0.85**	**0.04**	**0.09**	**1.11E-11**
	G1	-0.20	0.07	0.09	1.00E+00
	G2	-0.74	0.08	0.08	1.00E+00
	G3	-0.69	0.08	0.09	1.00E+00
	G6	-0.32	0.11	0.08	8.70E-05
	G4	-0.39	0.08	0.08	1.00E+00
	**G5**	**0.89**	**0.11**	**0.08**	**9.21E-07**
Tnfsf13b (Baff)	G0	2.11	0.07	0.07	1.00E+00
	**G1**	**1.19**	**0.14**	**0.05**	**4.76E-78**
	**G2**	**0.58**	**0.14**	**0.06**	**2.02E-23**
	G3	-1.04	0.06	0.07	1.00E+00
	G6	-1.90	0.04	0.07	5.15E-09
	G4	-1.83	0.02	0.07	2.14E-16
	G5	-1.24	0.03	0.07	2.47E-14
Cxcl13	G0	-3.06	0.04	0.31	1.59E-136
	G1	-0.39	0.21	0.29	1.18E-16
	G2	-1.26	0.22	0.29	1.65E-06
	**G3**	**1.88**	**0.53**	**0.22**	**0.00E+00**
	G6	-0.30	0.32	0.27	2.76E-01
	G4	-0.65	0.23	0.29	3.25E-03
	G5	-0.78	0.19	0.30	7.11E-36

A Wilcoxon ranked-sum test was used to compare expression of the indicated genes among CAF groups. *Avg_log2FC,* log2 fold change of gene expression by cells in the group of interest relative to cells outside the group of interest. *Pct.1,* percentage of expressing cells in the group of interest. *Pct.2,* percentage of expressing cells outside the group of interest. *P_val_adj,* p value adjusted using Bonferroni-Hochberg correction. Groups with statistically significant positive enrichment are in bold.

Supplementary Table 2 (included as .xlsx file): Identification of pathway terms enriched among CAF groups. Gene set pathway terms defined in the GO msigDB mouse collection selectively enriched in different CAF groups were identified as described in Methods. Qval represents the significance of enrichment for each pathway, while FC represents the negative or positive log2 fold change of a given pathway compared to all other groups. Signed_qval represents the qval multiplied by the sign of FC to indicate negative or positive enrichment. Terms enriched in each group are listed on individual sheets. Terms are sorted by signed_qval.

Supplementary Table 3 (included as .xlsx file): Differential expression of all genes among CAF groups. A Wilcoxon ranked-sum test was used to compare expression of all genes among CAF groups. *Avg_log2FC,* log2 fold change of gene expression by cells in the group of interest relative to cells outside the group of interest. *Pct.1,* percentage of expressing cells in the group of interest. *Pct.2,* percentage of expressing cells outside the group of interest. *P_val_adj,* p value adjusted using Bonferroni-Hochberg correction. Groups with statistically significant positive enrichment are shown in bold. Genes enriched in each group are listed on individual sheets. Genes are sorted by p_val_adj.

Supplementary Table 4 (included as .xlsx file): Expression of unique marker genes defined in human CAF single cell studies in CAF groups from the current study. Marker genes for the indicated CAF functional categories were compiled from references 23-27 and 93, and sorted based on Gene functional category. Markers unique to a particular CAF functional category are identified by color coding of the cells under each column. Expression of the corresponding gene in groups G0 through G6 is indicated by a colored cell containing a “+”. “Not included” indicates that the gene was not identified in any of the indicated references. “neg” indicates that the gene was not expressed at a detectable level in our analysis.

Supplementary Table 5. Number of previously defined unique genes expressed in groups from the current study

CAF functional category	Number of defined unique genes	G0	G1	G2	G3	G6	G4	G5
**ProCAF**	2	2	1	0	0	0	0	0
**Myofib**	8	1	4	3	2	1	2	0
**Matrix**	9	2	6	6	4	0	0	0
**Vascular**	2	0	0	0	0	0	0	0
**Inflammatory**	20	7	6	4	3	0	1	3
**Antigen presenting**	3	0	0	0	2	1	1	3
**Interferon**	3	1	1	1	0	0	0	1
**Reticular**	2	0	1	1	0	0	0	0
**Spatial S1/S2**	12	0	7	8	3	0	0	0
**Spatial S3**	9	0	3	3	3	0	1	0
**Spatial S4**	5	0	2	2	2	0	1	0

Data is a summary of information contained in **Supplementary Table 4**. Color coding indicates the minimum percentage of defined unique genes in each indicated functional category that are expressed by each group from the current study.

≥50% of unique marker genes expressed

≥33% of defined marker genes expressed

≥25% of defined marker genes expressed

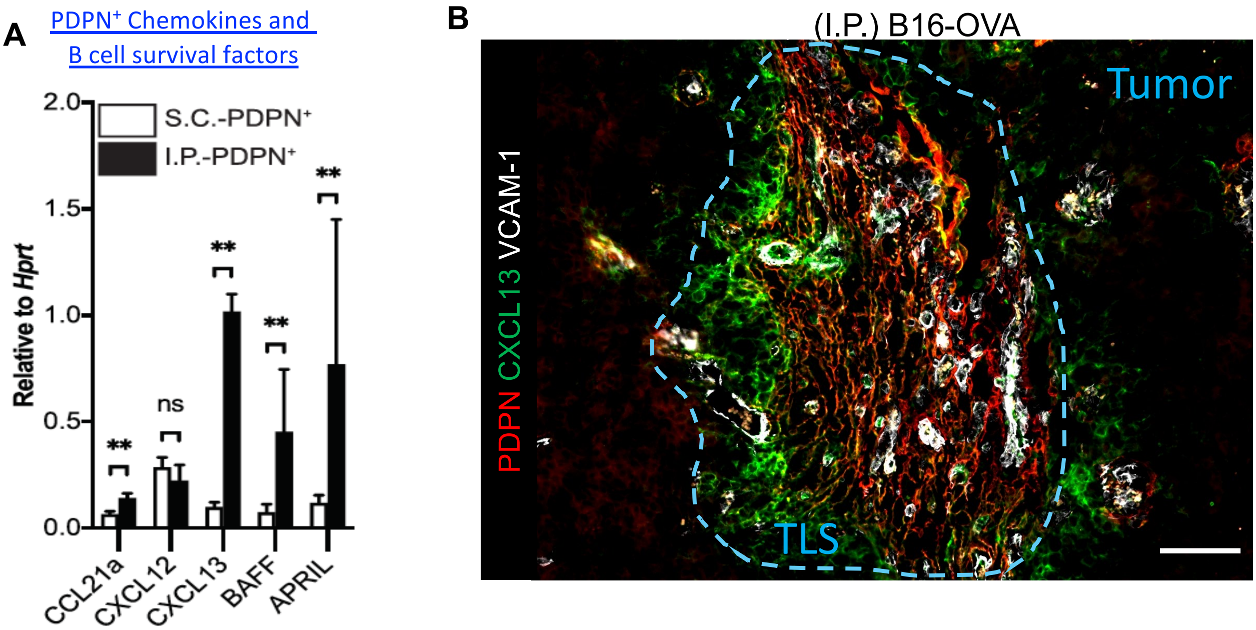

Supplementary Figure 1. CAF in IP tumors differentially express B cell organizing chemokine CXCL13 and B cell survival factors. (A) CAF from day 14 S.C. and I.P. tumors were enriched from CD45-depleted suspensions using biotin derivatized anti-PDPN and α-biotin magnetic beads (Miltenyi Biotec) on an AutoMACS Pro Separator (Miltenyi Biotec) according to the manufacturer's instructions. RNA was purified from whole tumor or sorted cells using RNAEasy kits (Qiagen) according to the manufacturer's instructions. High-Capacity cDNA Reverse Transcription Kit (Applied Biosystems) and purified RNA was used to generate cDNA. Amplification was performed using TaqMan Fast Advanced Master Mix (Applied Biosystems) and a QuantStudio 6 Flex Real-Time PCR system (Applied Biosystems) with the following program: 50°C for 2 minutes; 95°C for 2 minutes; 40 cycles of 95°C for 1 second, 60°C for 20 seconds. Data from 2 experiments is presented as $2^{-\Delta CT}$ relative to *Hprt*, n=6 tumors per group. *This figure was previously published by Cell Press under a Creative Commons BY-NC-ND license as Figure 3E in Rodriguez et. al., Cell Reports 36,109422 (2021).*

(B) Day 14 IP tumors were frozen, sectioned, and stained using antibodies specific for podoplanin (Biolegend #4749), VCAM-1 (AlexaFluor647, Biolegend #3129), and CXCL13 (biotinylated, R&D Systems baf470) + streptavidin (AlexaFluor488, Biolegend 9304) as described in Rodriguez et al. *Meth Mol Biol 1845*, 241-57 (2018). For the detection of intratumoral CAF, exposure times and thresholds were set to visualize bright PDPN+ cells located in perivascular and TA-TLS areas, while eliminating the dim tumor cell signal. All images were captured on an AxioImager with Apotome (Zeiss) and images were analyzed either by ImageJ Software (NIH) or Imaris image analysis software (Bitplane). For image presentation, brightness and contrast were linearly adjusted and color-merged images were generated using Photoshop CS6 Software (Adobe). Blue dashes delimit TA-TLS area. Scale bar = 100 μm. Image representative of two independent experiments, n=6.

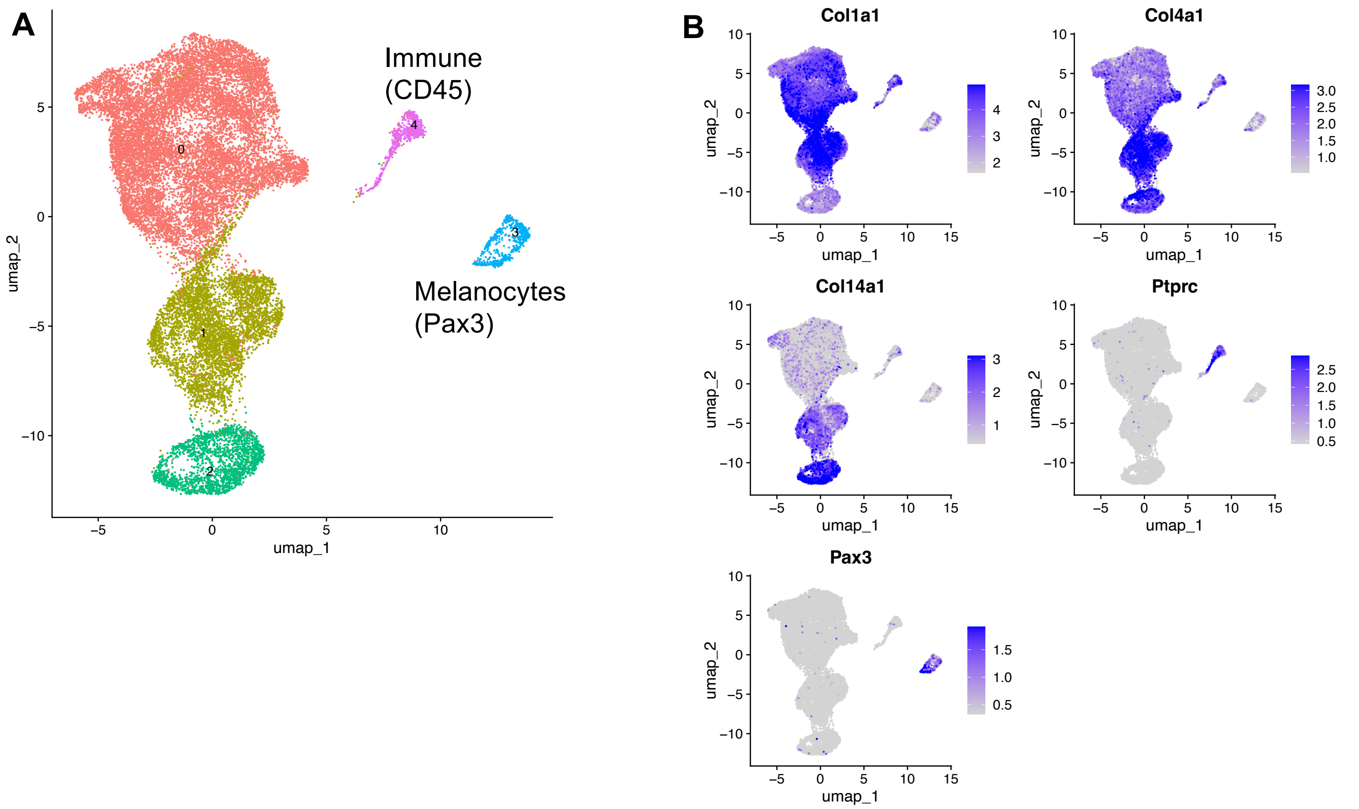

Supplementary Figure 2. Unsupervised clustering of PDPN-enriched populations from IP B16 melanomas. Single cell transcriptomic data of all cells passing quality control metrics was clustered by similarity in gene expression as described in Methods and plotted in a UMAP. (A) Distinct cell clusters are identified by color and number, with clusters 0, 1, and 2 representing fibroblasts, cluster 3 representing melanoma cells, and cluster 4 representing immune cells. (B) Distributions of select collagens, Pax3, and Cd45 (Ptprc) were used to identify fibroblast, melanoma, and immune cells, respectively.

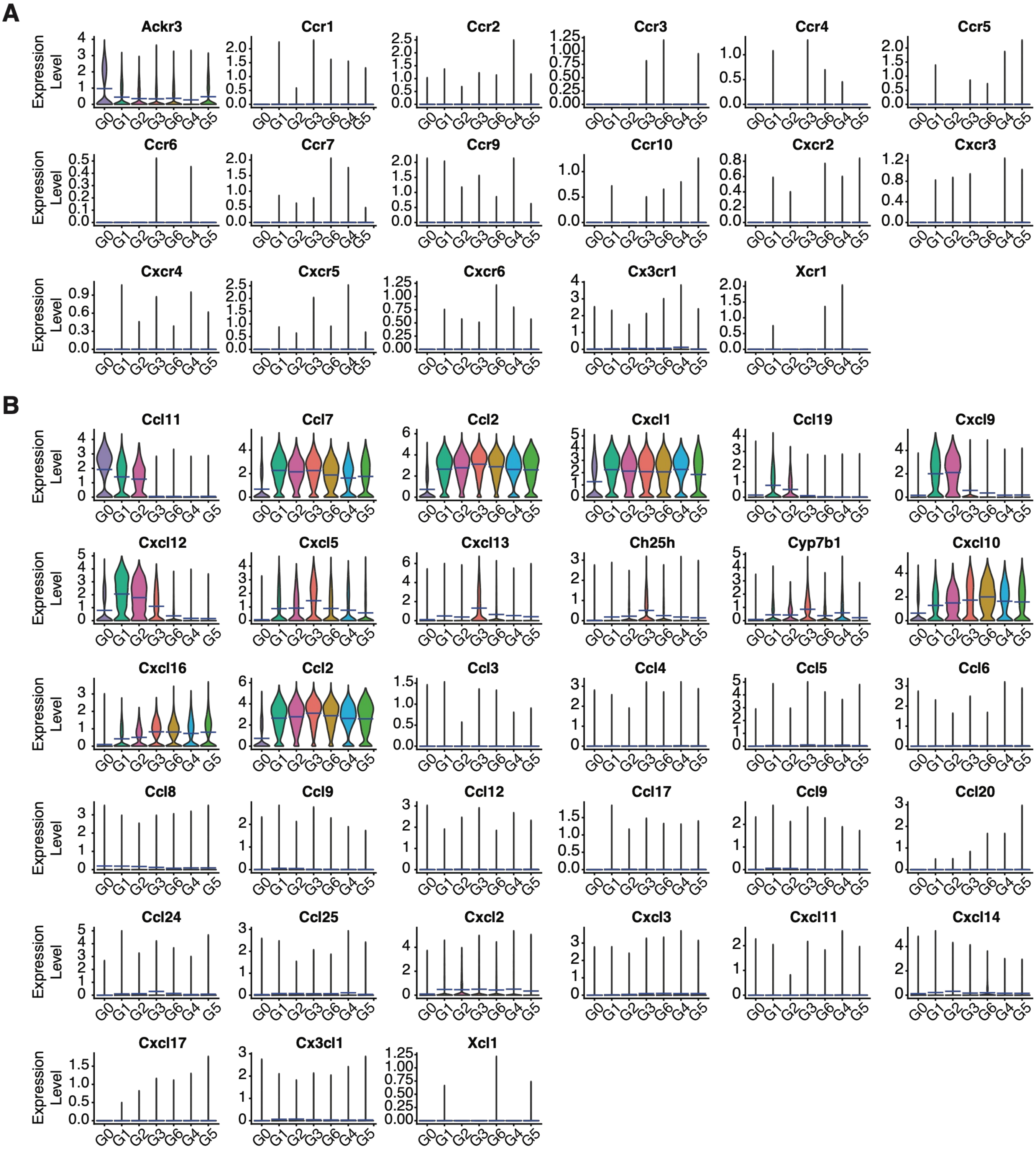

Supplementary Figure 3: Expression of chemokine receptor and chemokine encoding genes among CAF groups. Gene expression values for all (A) chemokine receptors and (B) chemokines that were expressed in at least one CAF are displayed by group in violin plots. Blue bars indicate mean expression value of the gene for each group. Genes showing statistically significant enrichment and percentage of expressing cells in at least one group are displayed first and included in **Figure 6**.

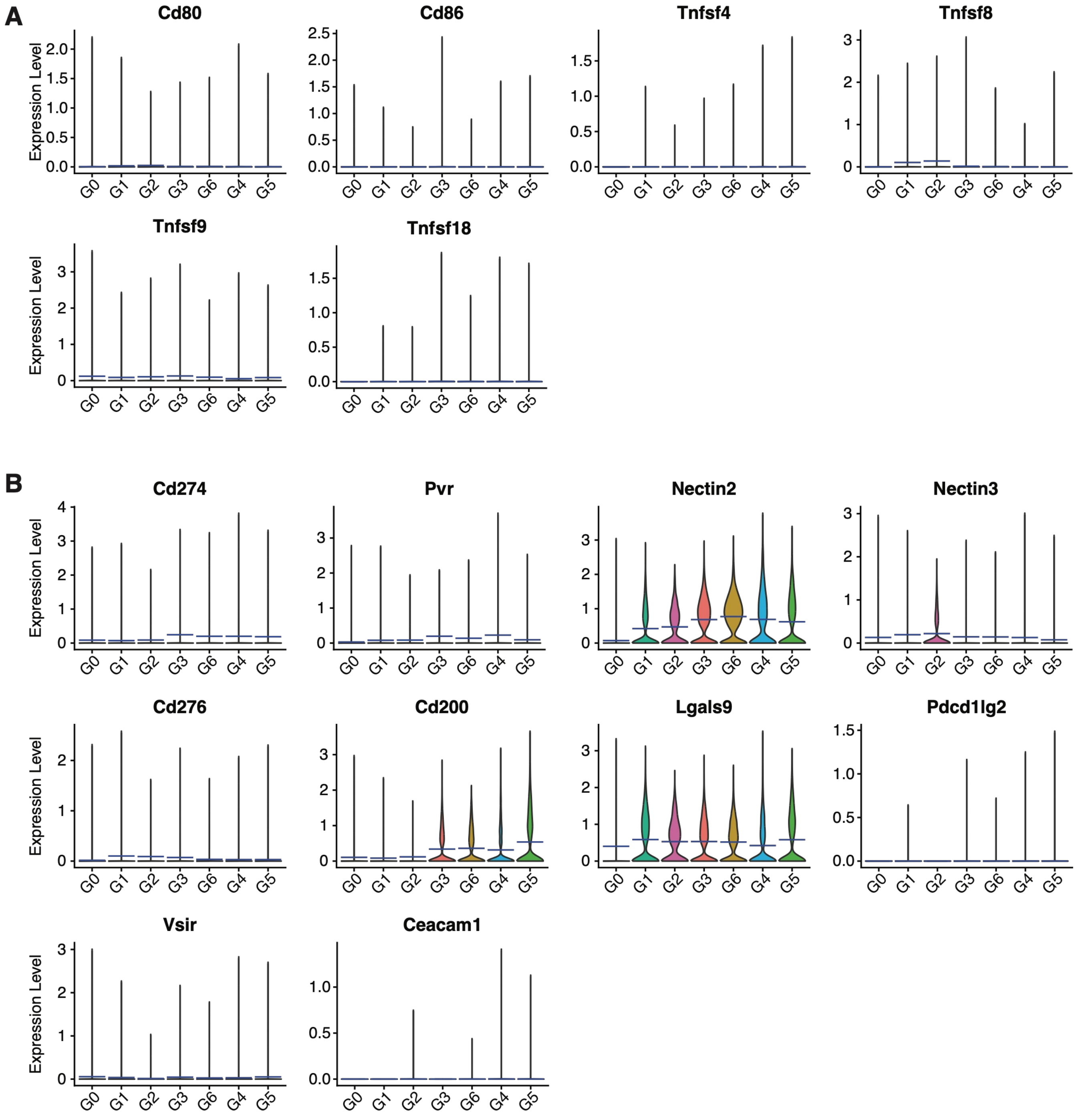

Supplementary Figure 4. Expression of genes associated with co-stimulation and inhibition among CAF groups. Gene expression values for all co-stimulatory (A) and inhibitory (B) ligands that were expressed in at least one CAF are displayed by group in violin plots. Blue bars indicate mean expression value of the gene for each group. (A) There was no detectable expression of *Tnfsf5 (Cd40L)* or *Tnfsf7 (Cd70).* Genes showing statistically significant enrichment and percentage of expressing cells in at least one group are displayed first and included in **Figure 7**.

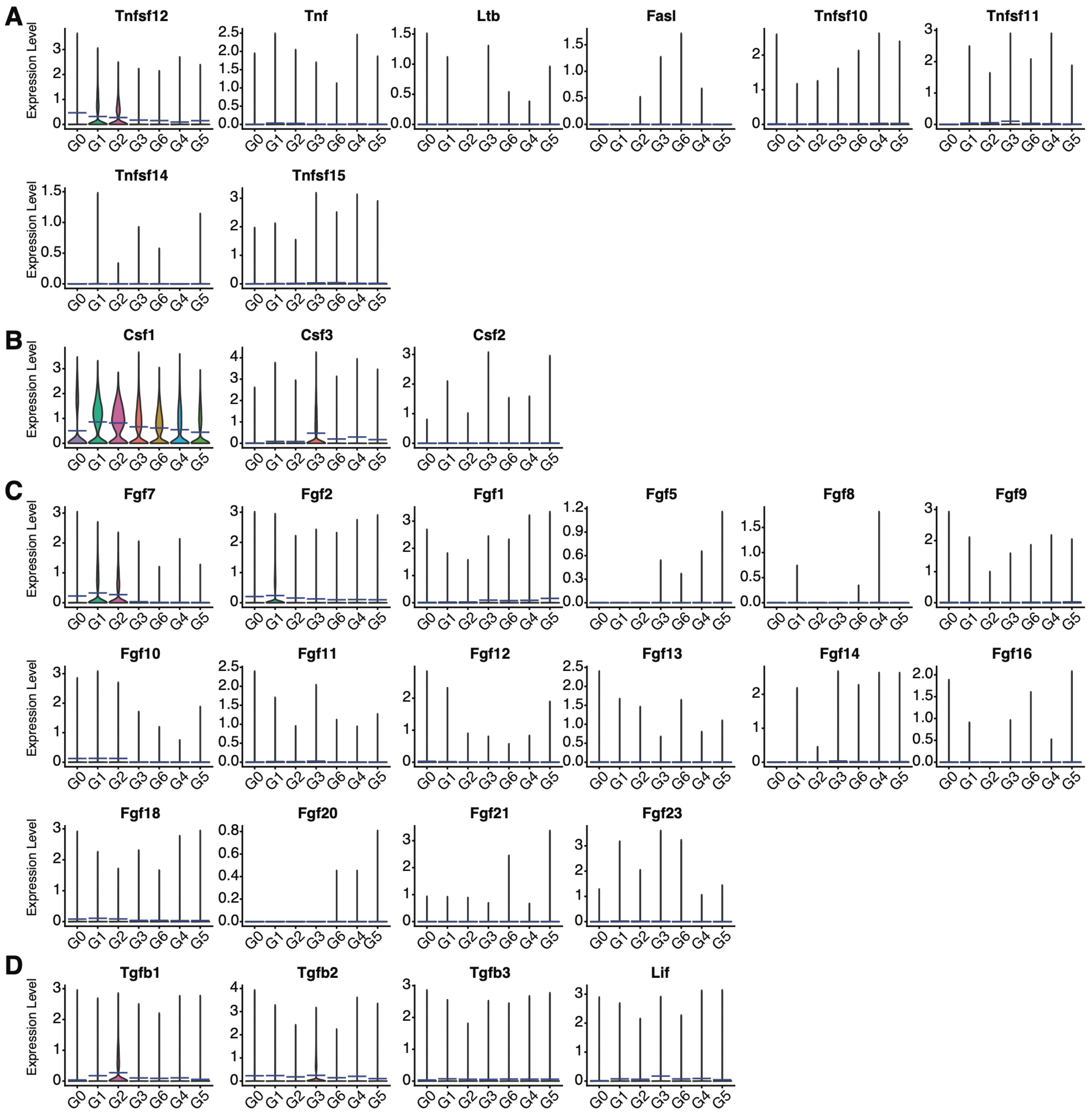

Supplementary Figure 5: Expression of cytokine genes by CAF groups. Gene expression values for all cytokines that were expressed in at least one CAF are displayed by group in violin plots. Blue bars indicate mean expression value of the gene for each group. (A) Soluble Tnfsf members excluding co-stimulatory molecules listed in Supplementary Figure 4. (B) Csfsf members. (C) Fgf sf members. (D) Tgf sf members. (E) Interleukins. (F) Interferons. Members of any group not shown were not detected in scRNA-seq data. Genes showing statistically significant enrichment and percentage of expressing cells in at least one group are displayed first and included in **Figure 7**.

E

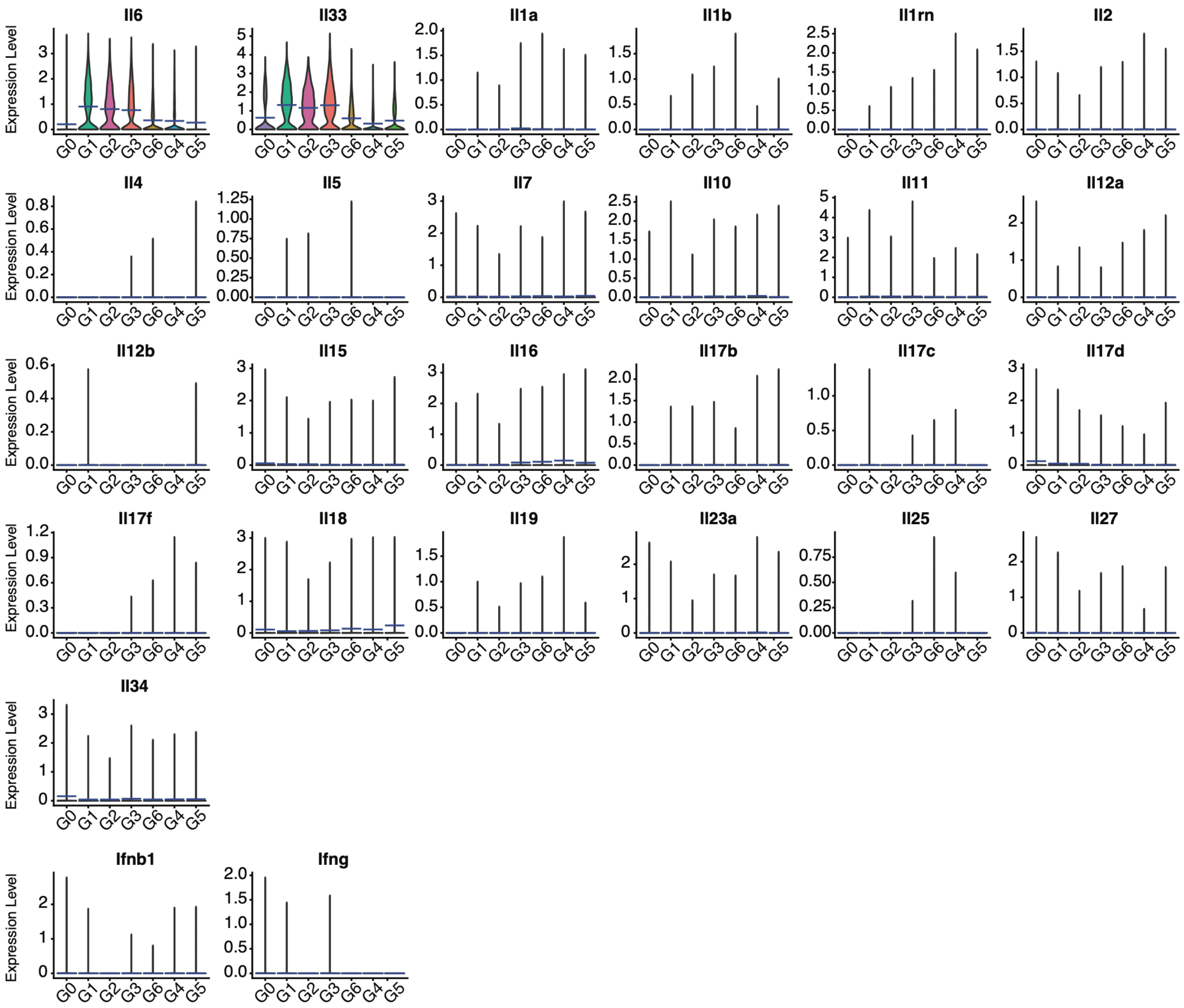

Supplementary Figure 5 (cont): Expression of cytokine genes by CAF groups. Gene expression values for all cytokines that were expressed in at least one CAF are displayed by group in violin plots. Blue bars indicate mean expression value of the gene for each group. (A) Soluble Tnfsf members excluding co-stimulatory molecules listed in Supplementary Figure 4. (B) Csfsf members. (C) Fgf sf members. (D) Tgf sf members. (E) Interleukins. (F) Interferons. Members of any group not shown were not detected in scRNA-seq data. Genes showing statistically significant enrichment and percentage of expressing cells in at least one group are displayed first and included in **Figure 7**.

A

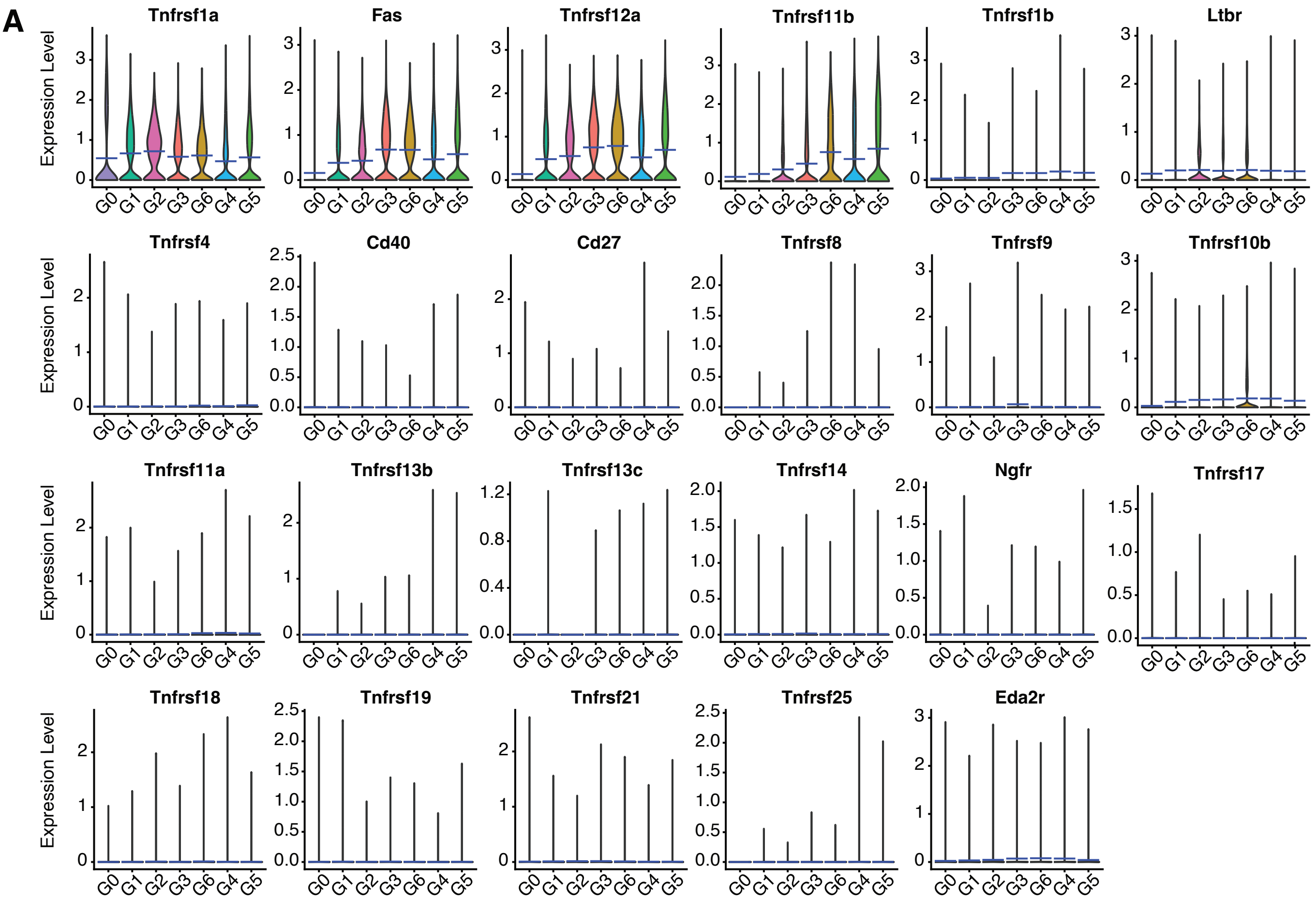

B

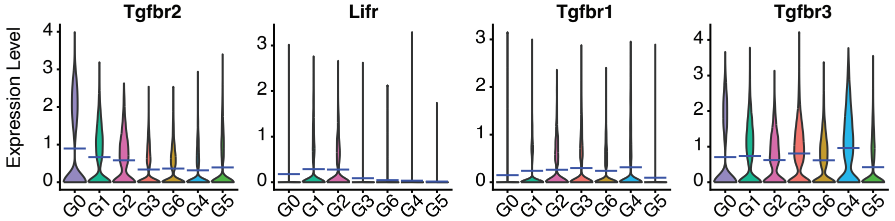

Supplementary Figure 6: Expression of cytokine receptor genes by CAF groups. Gene expression values for all cytokine receptors that were expressed in at least one CAF are displayed by group in violin plots. Blue bars indicate mean expression value of the gene for each group. (A) Tnfrsf members. (B) Tgfr sf members. (C) Interleukin receptors. (D) Interferon receptors. Members of any group not shown were not detected in scRNA-seq data. Genes showing statistically significant enrichment and percentage of expressing cells in at least one group are displayed first and included in **Figure 8**.

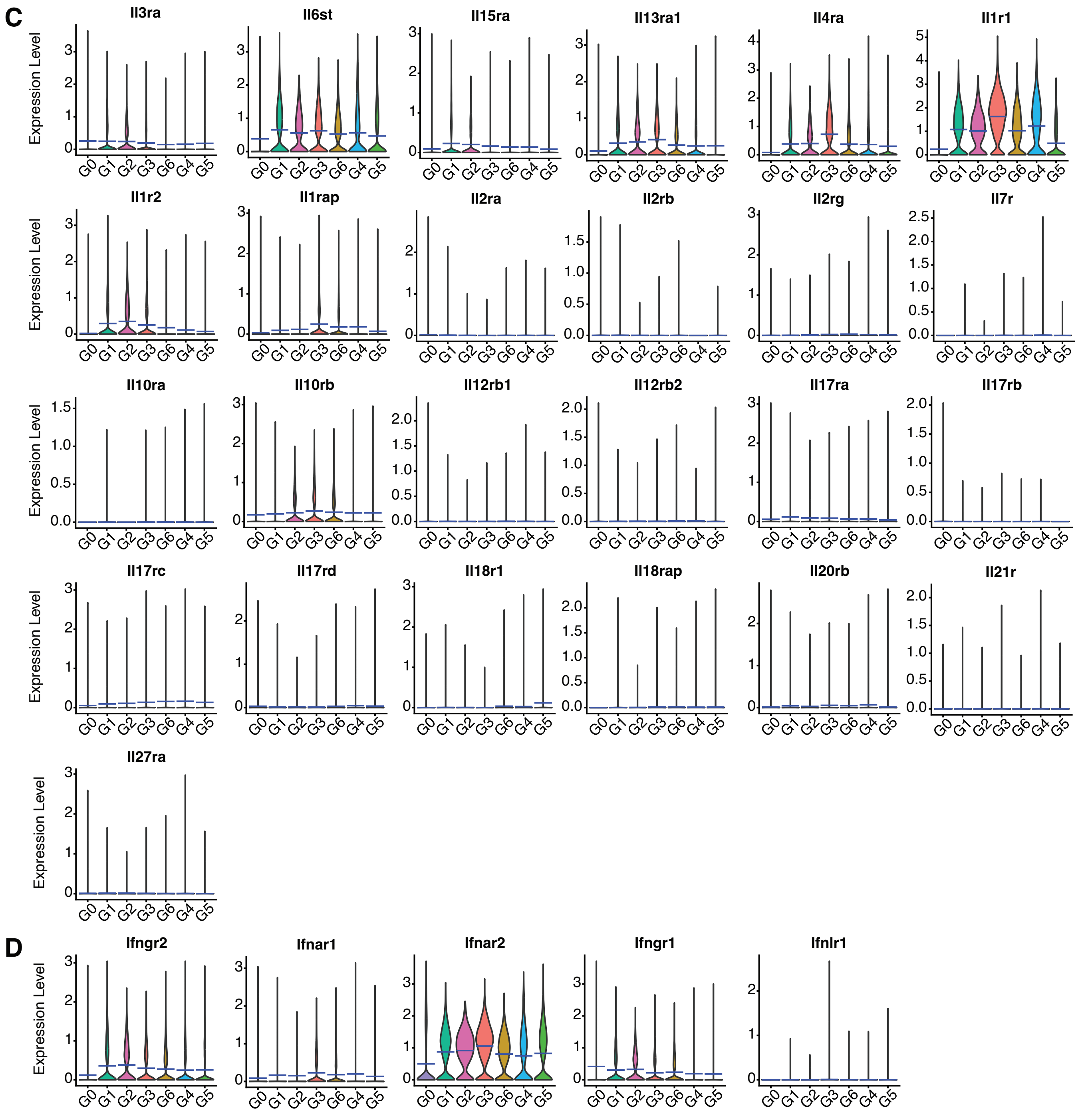

Supplementary Figure 6 (cont): Expression of cytokine receptor genes by CAF groups. Gene expression values for all cytokine receptors that were expressed in at least one CAF are displayed by group in violin plots. Blue bars indicate mean expression value of the gene for each group. (A) Tnfrsf members. (B) Tgfr sf members. (C) Interleukin receptors. (D) Interferon receptors. Members of any group not shown were not detected in scRNA-seq data. Genes showing statistically significant enrichment and percentage of expressing cells in at least one group are displayed first and included in **Figure 8**.

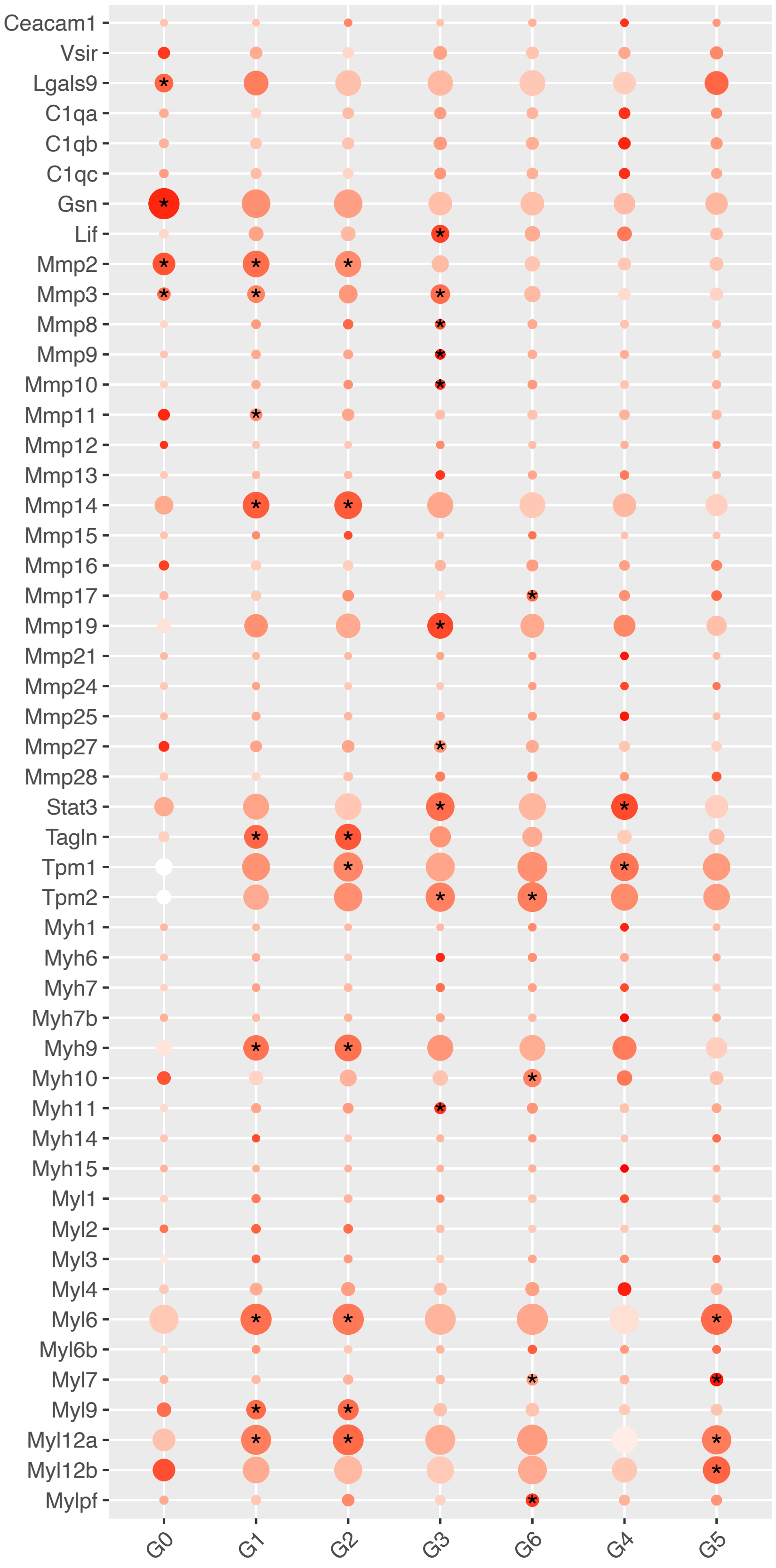

Supplementary Figure 7: Expression of additional genes in categories used to define CAF groups in other studies. (legend continues on next page**).**

Supplementary Figure 7: Expression of additional genes in categories used to define CAF groups in other studies. Genes were identified from references 23-27 and 93, as well as consideration of relevant functional categories (complement, matrix metalloproteinases, myosin heavy and light chains). For each gene, mean expression values for each group were normalized and shifted to establish a baseline of 0. Color intensity indicates normalized expression intensity. Dot size indicates percentage of cells positive for the gene by group. A Wilcoxon Ranked Sum test was run to identify genes significantly enriched in each group. Those marked for significance had a Bonferonni-Hochburg adjusted p value < 0.05, a positive log2 fold change > 0.25, and were expressed in $> 10\%$ of cells in the group in question. Data supporting this determination are shown in **Supplementary Table 3**.
